# Leucine deprivation results in antidepressant effects via GCN2 in AgRP neurons

**DOI:** 10.1093/lifemeta/load004

**Published:** 2023-02-04

**Authors:** Feixiang Yuan, Shangming Wu, Ziheng Zhou, Fuxin Jiao, Hanrui Yin, Yuguo Niu, Haizhou Jiang, Shanghai Chen, Feifan Guo

**Affiliations:** Zhongshan Hospital, State Key Laboratory of Medical Neurobiology, Institute for Translational Brain Research, MOE Frontiers Center for Brain Science, Fudan University, Shanghai 200032, China; Chinese Academy of Sciences (CAS) Key Laboratory of Nutrition, Metabolism and Food Safety, Innovation Center for Intervention of Chronic Disease and Promotion of Health, Shanghai Institute of Nutrition and Health, University of Chinese Academy of Sciences, Chinese Academy of Sciences, Shanghai 200031, China; Chinese Academy of Sciences (CAS) Key Laboratory of Nutrition, Metabolism and Food Safety, Innovation Center for Intervention of Chronic Disease and Promotion of Health, Shanghai Institute of Nutrition and Health, University of Chinese Academy of Sciences, Chinese Academy of Sciences, Shanghai 200031, China; Chinese Academy of Sciences (CAS) Key Laboratory of Nutrition, Metabolism and Food Safety, Innovation Center for Intervention of Chronic Disease and Promotion of Health, Shanghai Institute of Nutrition and Health, University of Chinese Academy of Sciences, Chinese Academy of Sciences, Shanghai 200031, China; Chinese Academy of Sciences (CAS) Key Laboratory of Nutrition, Metabolism and Food Safety, Innovation Center for Intervention of Chronic Disease and Promotion of Health, Shanghai Institute of Nutrition and Health, University of Chinese Academy of Sciences, Chinese Academy of Sciences, Shanghai 200031, China; Zhongshan Hospital, State Key Laboratory of Medical Neurobiology, Institute for Translational Brain Research, MOE Frontiers Center for Brain Science, Fudan University, Shanghai 200032, China; Chinese Academy of Sciences (CAS) Key Laboratory of Nutrition, Metabolism and Food Safety, Innovation Center for Intervention of Chronic Disease and Promotion of Health, Shanghai Institute of Nutrition and Health, University of Chinese Academy of Sciences, Chinese Academy of Sciences, Shanghai 200031, China; Zhongshan Hospital, State Key Laboratory of Medical Neurobiology, Institute for Translational Brain Research, MOE Frontiers Center for Brain Science, Fudan University, Shanghai 200032, China; Zhongshan Hospital, State Key Laboratory of Medical Neurobiology, Institute for Translational Brain Research, MOE Frontiers Center for Brain Science, Fudan University, Shanghai 200032, China

**Keywords:** leucine deprivation, depression, AgRP neurons, GCN2, amino acid sensing

## Abstract

Essential amino acids (EAAs) are crucial nutrients, whose levels change in rodents and patients with depression. However, how the levels of a single EAA affects depressive behaviors remains elusive. Here, we demonstrate that although deprivation of the EAA leucine has no effect in unstressed mice, it remarkably reverses the depression-like behaviors induced by chronic restraint stress (CRS). This beneficial effect is independent of feeding and is applicable to the dietary deficiency of other EAAs. Furthermore, the effect of leucine deprivation is suppressed by central injection of leucine or mimicked by central injection of leucinol. Moreover, hypothalamic agouti-related peptide (AgRP) neural activity changes during CRS and leucine deprivation, and chemogenetically inhibiting AgRP neurons eliminates the antidepressant effects of leucine deprivation. Finally, the leucine deprivation-regulated behavioral effects are mediated by amino acid sensor general control non-derepressible 2 (GCN2) in AgRP neurons. Taken together, our results suggest a new drug target and/or dietary intervention for the reduction of depressive symptoms.

## Introduction

Depression is a commonly diagnosed neuropsychiatric disease that severely limits psychosocial functioning and diminishes quality of life [1]. In addition, depression is associated with the development of serious health concerns, like abnormal food intake, type 2 diabetes, and sleep disorders [1, 2]. Its pathophysiology is complex and yet to be comprehensively elucidated [3]. While various antidepressant drugs have been introduced for clinical use, they are not always effective and have adverse effects [4, 5]. Hence, further investigation into the molecular pathophysiology of depression and the identification and testing of novel therapeutic approaches remain a necessity.

Nutrition is essential for the maintenance of normal emotional states. Unbalanced nutrition is implicated in the etiology of depression, potentially hindering treatment [6, 7]. For example, total protein intake is inversely associated with the risk of depressive symptoms [8]. Proteins are composed of amino acids (AAs), which are divided into essential amino acids (EAAs) and non-EAAs [9]. According to clinical studies, many EAAs in serum are changed in patients with depression, such as phenylalanine, methionine, tryptophan, threonine, and branched-chain amino acids (BCAAs), including leucine, isoleucine, and valine [10–13]. However, whether alterations in EAA levels contribute to depression and the underlying mechanisms remain largely unknown.

General control non-derepressible 2 (GCN2) is a key orchestrator of the stress response, modulating protein synthesis under conditions of AA starvation [14, 15]. It plays important roles in various physiological processes, such as angiogenesis, inflammation, and metabolism [16–18]. However, its involvement in depression remains elusive.

The hypothalamus is critical for nutrient sensing [19, 20]. The key region of this sensing network is the arcuate nucleus (ARC) of the hypothalamus, which contains two sets of important neurons, including orexigenic agouti-related peptide (AgRP) neurons, as well as anorexigenic proopiomelanocortin (POMC) neurons [21]. These neurons sense nutrient levels, as well as regulating energy intake and energy expenditure [22, 23]. Recently, these neurons have been implicated in depression-related behaviors [24, 25]. For example, activation of POMC neurons promotes stress-induced depression, while activation of AgRP neurons reverses stress-induced depressive behaviors [26, 27]. Whether these neurons, such as AgRP, are involved in AA regulation of depression is unknown.

Herein, we subjected mouse models of depression to a single EAA-deficient diet, notably, a leucine-deficient diet, which has been reported to regulate feeding, body weight, as well as glucose and lipid metabolism [28, 29]. We aimed to investigate the effect of EAA deprivation on depression-like behaviors. We found that short-term leucine deprivation had no effect under normal conditions, but it alleviated depressive behaviors in a chronic restraint stress (CRS)-induced model of depression in mice.

## Results

### Leucine deprivation does not affect behavior in depression tests under non-stress conditions

**Figure 1:**
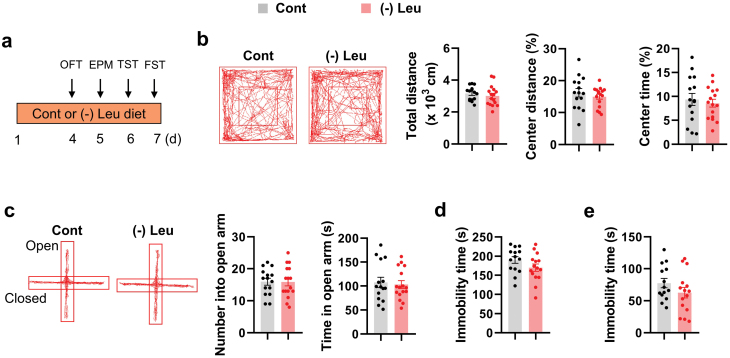
Short-term leucine deprivation does not cause depression-like behavior. (a) Timeline of the mice fed with control (Cont) or leucine-deficient [(-) Leu] diet and behavioral tests. (b) Representative tracks of mice in OFT, travel distance, percentage of distance in center area, and percentage of time spent in center. (c) Representative tracks of mice in EPM, the number of entries into the open arms, and the time spent in the open arms. (d and e) Immobility time of TST and FST, respectively. Studies were conducted using 8–9-week-old male WT mice fed a Cont or (-) Leu diet for 4−7 days. Data are expressed as the mean ± SEM (*n* = 14−16 per group, as indicated), with individual data points. Data were analyzed via two-tailed unpaired Student’s *t*-test.

To investigate whether lower leucine levels affected behaviors during depression tests, male C57BL/6J wild-type (WT) mice were given a control or leucine-deficient diet for 3 days and were then subjected to a panel of behavioral tests on days 4–7 (Fig. 1a). The experimental diet resulted in weight loss and decreased food intake compared with control mice (Supplementary Fig. S1a and b), as previously reported [29]. The anorexic signal, α-melanocyte-stimulating hormone (α-MSH) [30], increased in mice with leucine-deficient diet (Supplementary Fig. S1c). The locomotor activity measured in an open field test (OFT) was similar between groups (Fig. 1b). Further, the interest in exploring the central region in the OFT, the open arm region in the elevated plus maze test (EPM), as well as the immobility time in the tail suspension test (TST) and forced swim test (FST) were comparable between groups (Fig. 1b−e). A similar lack of difference in behaviors during such tests was also observed in female mice with leucine-deficient diet compared to female mice fed a control diet (Supplementary Fig. S1d−f). These results indicate that leucine deprivation has no effect on behavior in various depression tests under normal conditions.

### Leucine deprivation relieves CRS-induced depression-like behaviors

Because leucine deprivation did not affect any behaviors during depression testing under normal conditions, we further explored its effects in a mouse model of depression. First, we employed CRS to induce depression-related behaviors [31]. Male WT mice were subjected to a 3-h restraint (13:00–16:00) every day for 18 consecutive days, while the control mice were kept in cages unrestrained, as normal, but were also not provided food or water during the same 3-h period (Supplementary Fig. S2a). After 18 days, CRS mice exhibited reduced body weight but no change in food intake relative to that of control mice (Supplementary Fig. S2b and c). CRS mice displayed reduced center time and distance in OFT, decreased time in open arms in the EPM, and increased immobility time in TST, as well as FST, compared to that of control mice (Supplementary Fig. S2d–g). Though the hypothalamic-pituitary-adrenal (HPA) axis is related to stress [32], no obvious changes in the HPA axis were observed after CRS compared to the unrestrained control mice, as evaluated by examining the mRNA levels of corticotropin-releasing hormone, and the hormone levels of adrenocorticotropic hormone (ACTH) and corticosterone [33–35] (Supplementary Fig. S2h–j).

**Figure 2:**
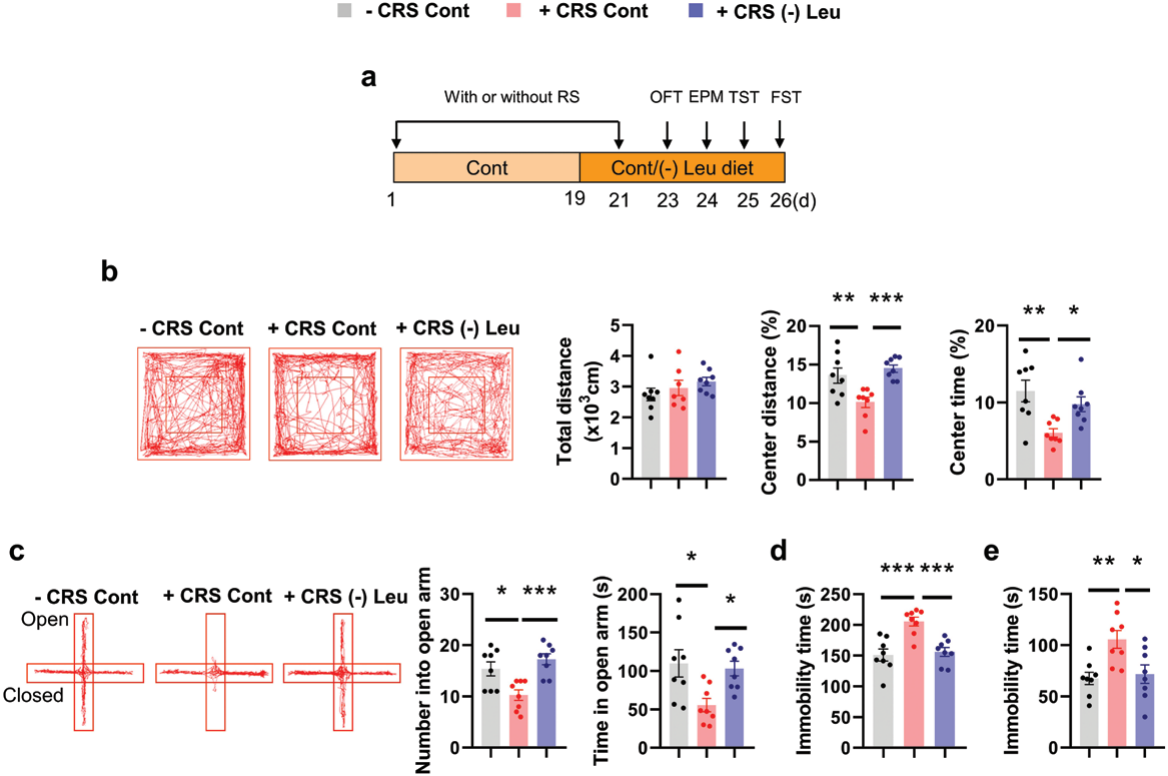
Leucine deprivation relieves CRS-induced depressive behaviors. (a) Schematic diagram illustrating the timeline of the restraint or control protocol and behavioral tests. (b) Representative tracks of mice in the OFT, travel distance, percentage of distance in center area, and percentage of time spent in center. (c) Representative tracks of mice in the EPM, the number of entries into the open arms, and the time spent in the open arms. (d and e) Immobility time in the TST and FST, respectively. Studies were conducted using 8–9-week-old male WT mice with or without CRS fed a Cont or (-) Leu diet for 4–7 days. Data are expressed as the mean ± SEM (*n* = 8 per group, as indicated), with individual data points. Data were analyzed via one-way ANOVA followed by the Student–Newman–Keuls (SNK) test. *P < 0.05, **P < 0.01, ***P < 0.001.

Next, we conducted the leucine deprivation experiments under CRS. Two days before the end of CRS, the mice were fed a control diet or leucine-deficient diet, followed by behavioral testing (Fig. 2a). As previously reported [2], CRS mice exhibited a lower interest in exploring the central region in OFT and the open arm region in EPM (Fig. 2b and c). Further, we observed greater despair in CRS mice, as indicated by the increased immobility in TST and FST (Fig. 2d and e). Surprisingly, CRS mice fed the leucine-deficient diet had increased center time and center distance in OFT, increased open arm time and entry in the EPM, as well as reduced immobility time in TST and FST, when compared with CRS mice fed a control diet (Fig. 2b–e). Leucine deprivation also induced weight loss and lower food intake during CRS (Supplementary Fig. S3a and b), in line with previous reports under normal conditions [29]. The alleviation of depression-like behaviors was also observed in female mice with leucine-deficient diet under CRS (Supplementary Fig. S3c).

To investigate whether shorter leucine deprivation could also have beneficial effects, we conducted 1- and 3-day leucine-deficient diet feeding following CRS exposure. One day of leucine-deficient diet feeding had no effects on TST results, while 3 days of leucine deprivation reduced the immobility time in the TST after CRS (Supplementary Fig. S3d and e). To investigate how long the antidepressant effect of leucine deprivation lasts, we returned the CRS mice to a control diet after feeding them a leucine-deficient diet for 7 days. After 3 days or 7 days of returning to a control diet, the CRS mice that had been treated with a leucine-deficient diet still exhibited reduced immobility time in TST, but the effect disappeared after 10 days of returning them to a control diet (Supplementary Fig. S3f−h).

To determine whether a leucine-deficient diet could attenuate depressive behaviors during CRS, male WT mice were subjected to CRS for 18 consecutive days, and the mice were fed with control or leucine-deficient diet on day 12 of CRS, followed by behavioral tests (Supplementary Fig. S4a). During CRS, the mice fed a control diet and fed a leucine-deficient diet exhibited similar behaviors in OFT, EPM, and TST (Supplementary Fig. S4b−f). These results suggest a leucine-deficient diet cannot attenuate depressive behaviors during CRS.

To determine the contribution of feeding-associated effects during leucine deprivation, we conducted pair-feeding experiments, which involved feeding CRS mice the same weight of control diet as the weight eaten by the leucine-deficient diet-fed group exposed to CRS (Supplementary Fig. S5a). In other words, the pair-feeding regimen with a control diet mimics the reduced food intake induced by leucine-deficient diet feeding while avoiding any direct effects of leucine deficiency. We found that the pair-fed mice had a lower body weight compared to that of CRS mice, which was still heavier than the leucine-deficient diet-fed group (Supplementary Fig. S5b). Importantly, the CRS-exposed pair-fed mice exhibited similar behaviors as CRS mice on the control diet, with no reversal of the depression-like behaviors, such as those observed in the leucine-deficient diet-fed mice, as determined via OFT, EPM, and TST (Supplementary Fig. S5c–e).

To assess whether the beneficial effects against CRS-induced depression were leucine-specific, we conducted analogous experiments focusing on seven other EAAs, including isoleucine, valine, lysine, methionine, phenylalanine, threonine, and tryptophan. Similar to the effects of a leucine-deficient diet, all mice given different single EAA-deficient diets had increased open arm time and entry in EPM, and decreased immobility time in TST (Supplementary Fig. S6). These results show that the beneficial effects of single AA deprivation can be extended to all EAAs and that, at least for leucine-deficient diet feeding, the effect is independent of feeding behavior.

### Intracerebroventricular (ICV) injection of leucine blocks leucine deprivation-induced antidepressant behaviors

**Figure 3:**
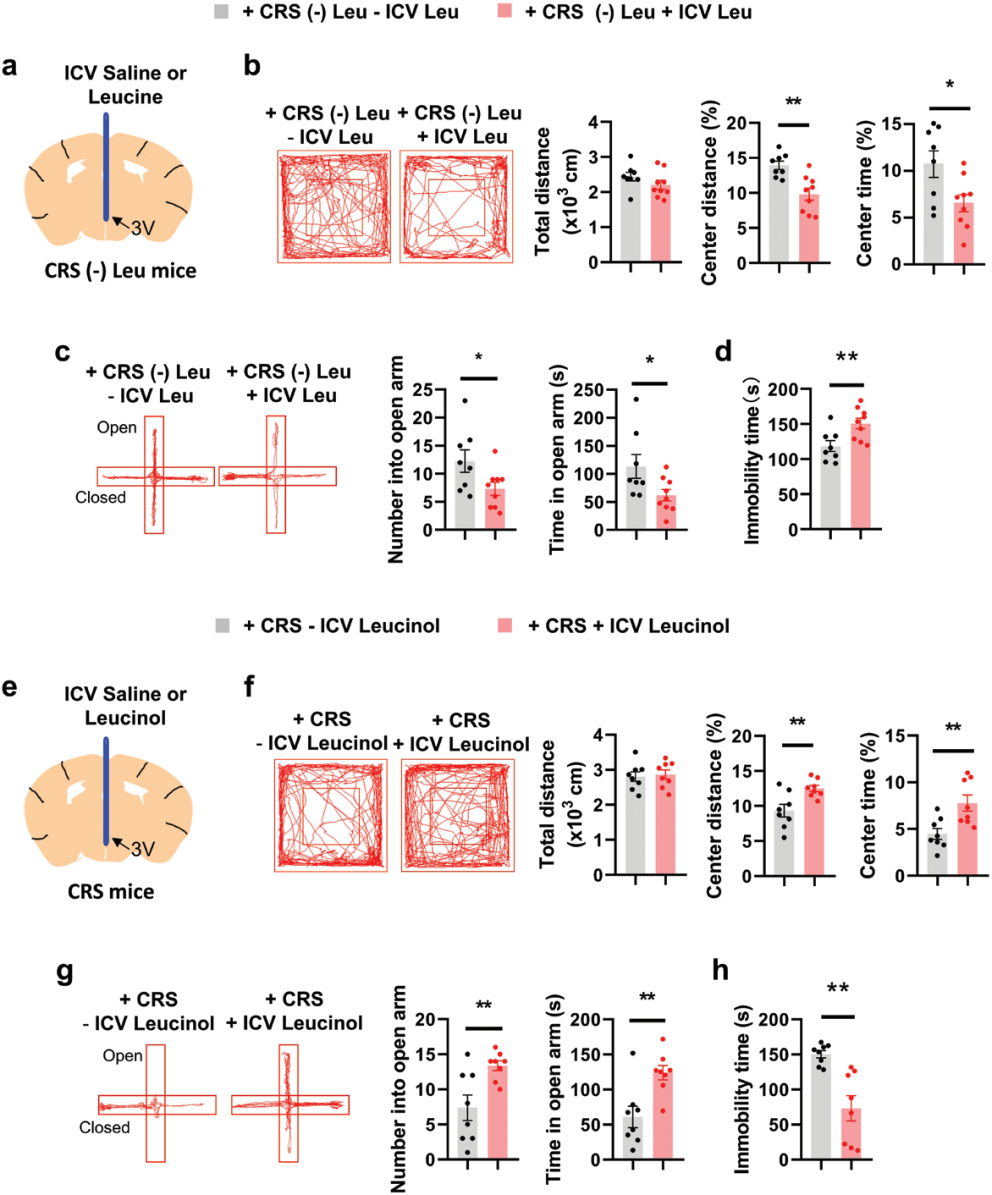
Effects of ICV injection of leucine and leucinol on depressive behaviors. (a and e) Schematic diagram illustrating the ICV injection of leucine or leucinol in mice with CRS fed a Cont or a (-) Leu diet. (b and f) Representative tracks of mice in OFT, travel distance, percentage of distance in center area, and percentage of time spent in center. (c and g) Representative tracks of mice in the EPM, the number of entries into the open arms, and the time spent in the open arms. (d and h) Immobility time of the TST. Studies for a–d were conducted using 7–8-week-old female WT mice with CRS fed a (-) Leu diet with ICV of saline (− ICV Leu) or leucine (+ ICV Leu) for 4–7 days; studies for e–h were conducted using 7–8-week-old female WT mice with CRS fed a Cont diet with ICV of saline (− ICV Leucinol) or leucinol (+ ICV Leucinol) for 4−7 days. Data are expressed as the mean ± SEM (*n* = 8–9 per group, as indicated), with individual data points. Data were analyzed via two-tailed unpaired Student’s *t*-test. *P < 0.05, **P < 0.01.

As previous studies reported that the hypothalamus directly senses AAs [29, 36], we questioned whether this brain region could indeed sense AA levels to regulate depression-related behaviors. Hypothalamic leucine levels were decreased after leucine-deficient diet feeding (data not shown), as previously reported [29]. Hence, we sought to determine whether hypothalamic leucine supplementation could reverse leucine-deficient diet-induced antidepressant effects. After CRS, ICV injection of leucine decreased center time and center distance in OFT, decreased open arm time and entry in EPM, and increased the immobility time in TST when compared to that of control mice under leucine deprivation, without affecting body weight or food intake, both in male and female mice (Fig. 3a−d, Supplementary Fig. S7a−f). However, ICV injection of leucine had no effect on behavioral tests after CRS without leucine deprivation (Supplementary Fig. S7g−j).

To investigate whether hypothalamus-specific leucine deprivation could induce antidepressant effects, we injected leucinol to the third cerebral ventricle of mice using a cannula (Fig. 3e) in order to mimic leucine deprivation in hypothalamus [36]. Leucinol is known to increase the intracellular level of uncharged Leu-tRNA by inhibiting Leucyl-tRNA synthetase, thus mimicking leucine deprivation [14]. After CRS, ICV injection of leucinol induced no change of body weight or food intake (Supplementary Fig. S8a and b). ICV injection of leucinol increased center time and center distance in OFT, increased open arm time and entry in EPM, and reduced immobility time in TST compared to that of control mice after CRS, both in male and female mice (Fig. 3f−h, Supplementary Fig. S8c−f). These results suggest that hypothalamic leucine is crucial for affecting CRS-induced depression-like behaviors.

### The beneficial role of leucine deprivation on CRS-induced depression is dependent on AgRP neurons

**Figure 4:**
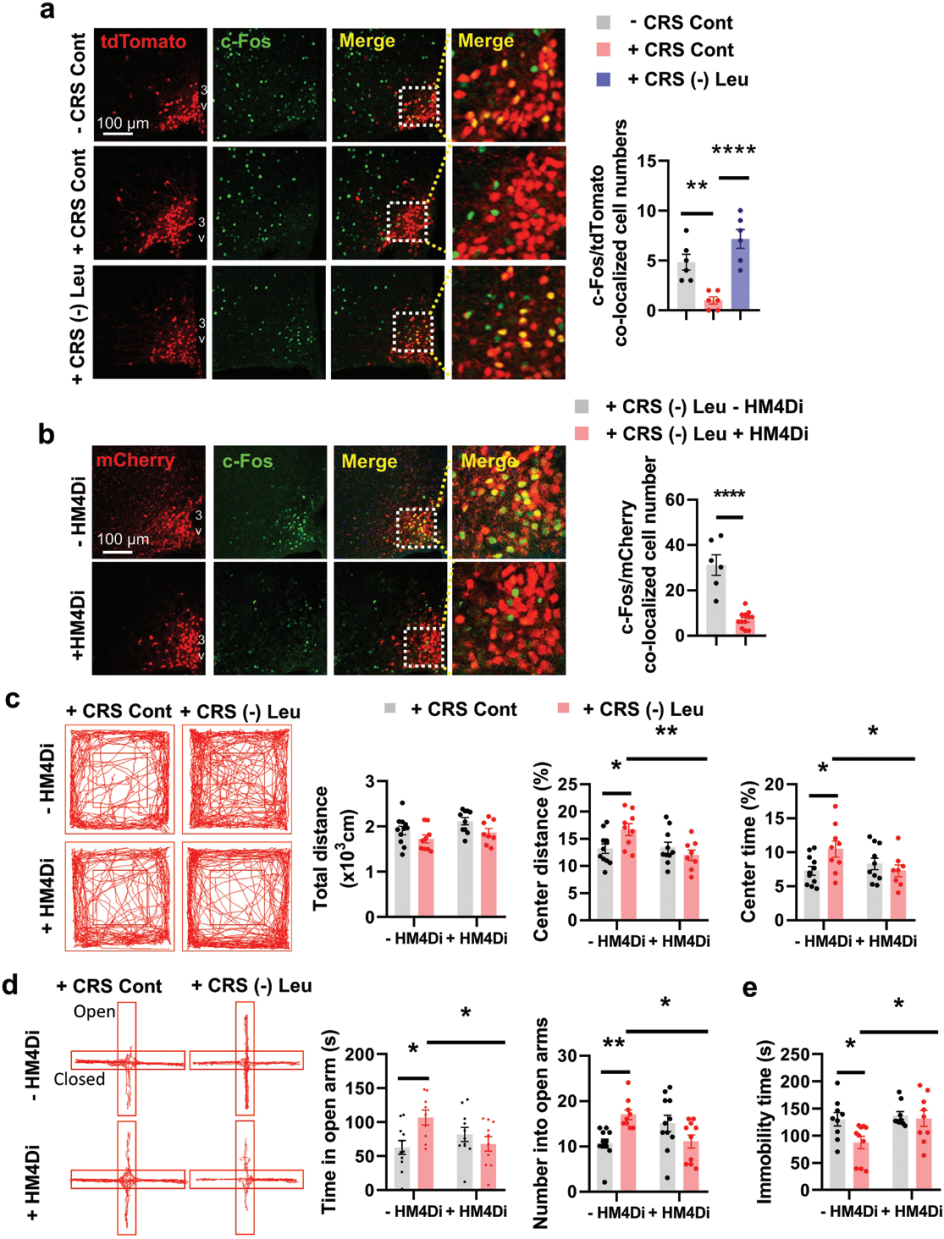
Inhibition of AgRP neural activity blocks leucine deprivation-induced antidepressant effects. (a) IF staining for tdTomato (red), c-Fos (green), and merge (yellow) in ARC (left), and quantification of c-Fos and tdTomato colocalized cell numbers (right); 3V, third ventricle. (b) IF staining for mCherry (red), c-Fos (green), and merge (yellow) in ARC (left), and quantification of c-Fos and mCherry colocalized cell numbers (right). (c) Representative tracks of mice in the OFT, travel distance, percentage of distance in center area, and percentage of time spent in center. (d) Representative tracks of mice in the EPM, the number of entries into the open arms, and the time spent in the open arms. (e) Immobility time in the TST. Studies for a were conducted using 8–9-week-old female AgRP-Ai9 mice with or without CRS fed a Cont or (-) Leu diet for 4–7 days; studies for b–e were conducted using 8–16-week-old male AgRP-Cre mice receiving AAVs expressing mCherry (− hM4Di) or hM4Di (+ hM4Di) under CRS and fed a Cont or (-) Leu diet for 4–7 days. Data are expressed as the mean ± SEM (*n* = 6–11 per group, as indicated), with individual data points. Data were analyzed via one-way ANOVA followed by the SNK test for a, or via two-tailed unpaired Student’s *t*-test for b, or via two-way ANOVA for c–e. *P < 0.05, **P < 0.01, ****P < 0.0001.

As hypothalamic leucine and leucinol are crucial for depression-like behaviors, we speculated that neurons within the hypothalamus have relevant roles. Previous work has shown that hypothalamic AgRP neurons are involved in the regulation of depression-related behaviors under chronic stress [27]. We sought to determine whether AgRP neurons were involved in the beneficial effect of leucine deprivation on depressive behaviors by performing immunofluorescence (IF) staining to examine the change of c-Fos, a signal reflecting neuronal activity [37]. IF staining revealed that c-Fos expression in AgRP neurons was decreased under CRS and increased after leucine deprivation, both in male and female mice (Fig. 4a and Supplementary Fig. S9a). IF staining of two additional signals reflecting neuronal activity [38, 39], FosB and phosphorylated extracellular signal-regulated kinase (p-ERK), resulted in similar changes under leucine deprivation after CRS (Supplementary Fig. S9b and c). Several animal models related to CRS and EAA deficiency tested above, including ICV-delivered leucine and leucinol treatments and feeding with other EAA deficient diets, also had coincident c-Fos changes in AgRP neurons (Supplementary Fig. S9d−f). In addition, the gene expression of *Agrp* and *Npy*, whose protein products (AgRP and neuropeptide Y respectively) are secreted from AgRP neurons, also increased after leucine deficiency under CRS (Supplementary Fig. S9g and h). The c-Fos expression in AgRP neurons also increased in mice after leucine deprivation in non-stressed mice (Supplementary Fig. S9i).

We then investigated the effect of chemogenetic inhibition of AgRP neural activity on leucine deprivation-induced anti-depressive behaviors in the CRS model. To this end, we employed inhibitory hM4Di designer receptors exclusively activated by designer drugs (DREADDs), which are activated by the inert ligand clozapine *N-oxide* (CNO) [37]. A Cre-dependent AAV encoding hM4Di (AAV-DIO-hM4Di-mCherry) or mCherry (AAV-DIO-mCherry) was injected into the ARC of AgRP-Cre male mice, and all these mice were then intraperitoneally (i.p.) injected with CNO before experiments (Supplementary Fig. S10a). The inhibition of AgRP neural activity was then confirmed based on the reduced IF staining of c-Fos in AgRP neurons in mice injected with hM4Di (Fig. 4b), in addition to reduced food intake after fasting, as AgRP neurons are required for the refeeding response [40] (Supplementary Fig. S10b). Inhibiting the neuronal activity of AgRP neurons largely blocked leucine deprivation-induced anti-depressive effects under CRS, as indicated by OFT, EPM, and TST results (Fig. 4c−e), without affecting body weight or daily food intake (Supplementary Fig. S10c and d). Similar results were found in female mice with AgRP neuron inhibition (Supplementary Fig. S11). These results show that the beneficial effects of leucine deprivation are mediated via activation of AgRP neurons.

### GCN2 in AgRP neurons mediates leucine deprivation-induced antidepressant effects during CRS

As the hypothalamus can directly sense AA levels [29, 36], we hypothesized that AgRP neurons might be able to sense leucine deprivation via the AA sensor GCN2 [14, 41] and increase its activity in response. To assess whether GCN2 was involved in the regulation of the antidepressant effects of leucine deprivation, we first assessed depression-like behaviors in global GCN2 knockout mice [17]. The knockout efficiency was validated based on GCN2 mRNA and protein levels in the hypothalamus and liver (Supplementary Fig. S12a and b). The leucine deprivation-induced beneficial behaviors were blocked in global GCN2 knockout mice under CRS, as indicated by OFT, EPM, and TST results (Supplementary Fig. S12c−e). Body weight and food intake were similar between the two groups (Supplementary Fig. S12f −k).

**Figure 5:**
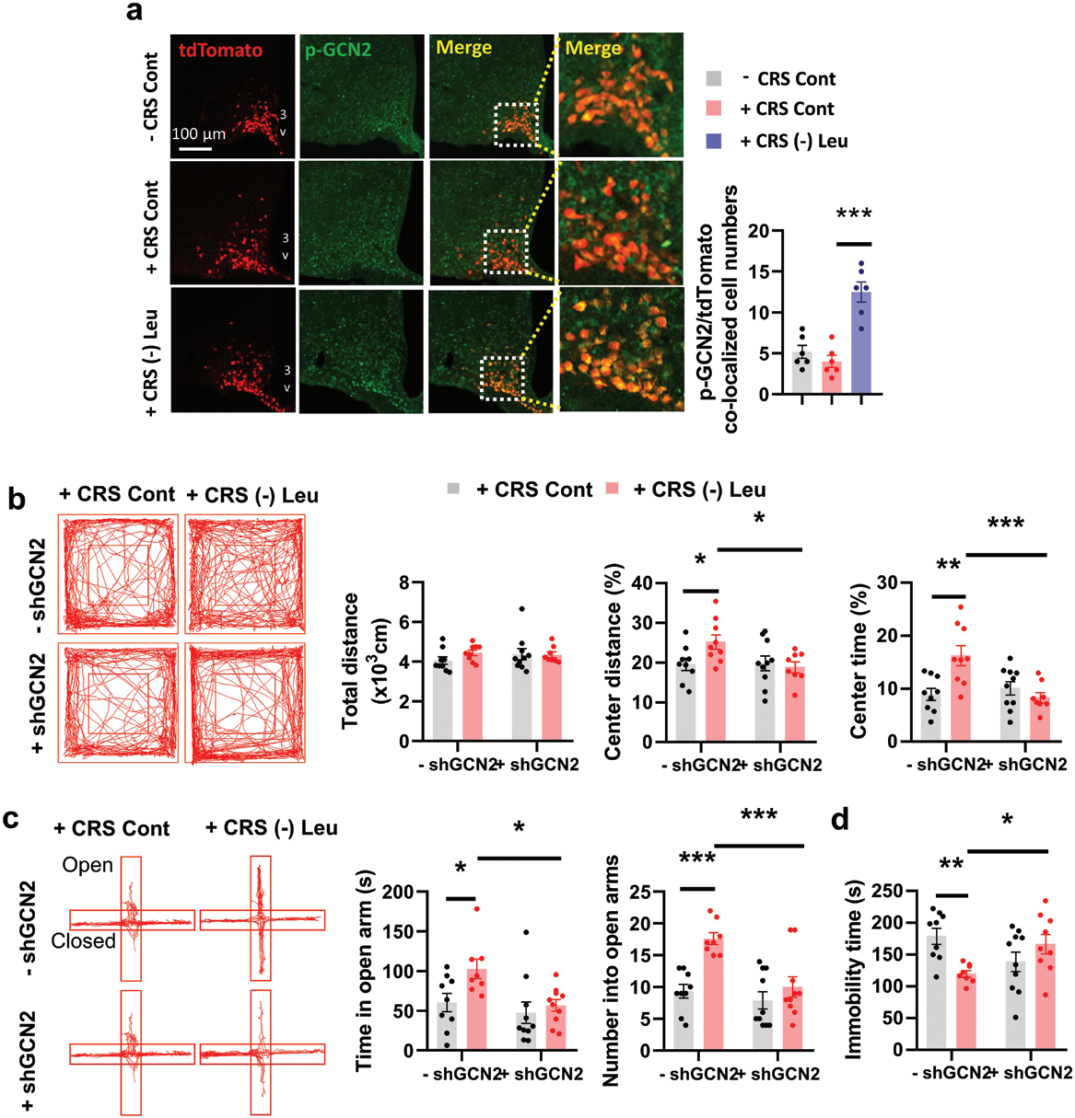
Knockdown of GCN2 in AgRP neurons disrupts leucine deprivation-induced antidepressant effects. (a) IF staining for tdTomato (red), p-GCN2 (green), and merge (yellow) in ARC (left), and quantification of p-GCN2 and tdTomato colocalized cell numbers (right); 3V, third ventricle. (b) Representative tracks of mice in the OFT, travel distance, percentage of distance in center area, and percentage of time spent in center. (c) Representative tracks of mice in the EPM, the number of entries into the open arms, and the time spent in the open arms. (d) Immobility time in the TST. Studies for a were conducted using 8–9-week-old female AgRP-Ai9 mice with or without CRS fed a Cont or (-) Leu diet for 4–7 days; studies for b–d were conducted using 10–12-week-old female AgRP-Cre mice receiving AAVs expressing GFP (− shGCN2) or shGCN2 (+ shGCN2) with CRS fed a (-) Leu diet for 4–7 days. Data are expressed as the mean ± SEM (*n* = 6–10 per group, as indicated), with individual data points. Data were analyzed via one-way ANOVA followed by the SNK test for a, or via two-way ANOVA for b–d. *P < 0.05, **P < 0.01, ***P < 0.001.

We then tested whether GCN2 in AgRP neurons also plays a critical role in the beneficial effects of leucine deprivation on depressive behaviors in the CRS model. IF staining revealed that the levels of p-GCN2 in AgRP neurons was unchanged after CRS, but increased after leucine deprivation (Fig. 5a). We then investigated whether knockdown of GCN2 in AgRP neurons would block the leucine deprivation-induced antidepressant effects during CRS. To this end, Cre-dependent AAVs encoding a short hairpin RNA directed against GCN2 (AAV-Flex-shGCN2-GFP) or GFP (AAV-Flex-GFP) were injected into the ARC of AgRP-Cre mice (Supplementary Fig. S13a), which allows the AAV vector to express desired genes and GFP proteins only in AgRP neurons, by the Cre-Flex system [42]. IF staining of GFP (reflecting AgRP neurons) and GCN2 revealed that GCN2 was colocalized with AgRP neurons in control mice, but this colocalization was significantly reduced in AgRP-shGCN2 mice (Supplementary Fig. S13b). The mRNA expression of *Gcn2* in the ARC was also lower in AgRP-shGCN2 mice compared to mice on the same diet but without knockdown of GCN2 (Supplementary Fig. S13c). However, GCN2 expression remained unchanged outside of AgRP neurons in the ARC for all mice (Supplementary Fig. S13b). Under leucine deprivation and CRS, the body weight and food intake of AgRP-shGCN2 mice were like those of control mice (Supplementary Fig. S13d−i). Moreover, knockdown of GCN2 in AgRP neurons blocked leucine deprivation-induced antidepressant effects under CRS, as indicated by changes in OFT, EPM, and TST (Fig. 5b–d). These results suggest that GCN2 in AgRP neurons is crucial for the beneficial effect of leucine deprivation on depressive behaviors in the CRS model.

### AgRP neuron activation reverses the effects of GCN2 knockdown

**Figure 6:**
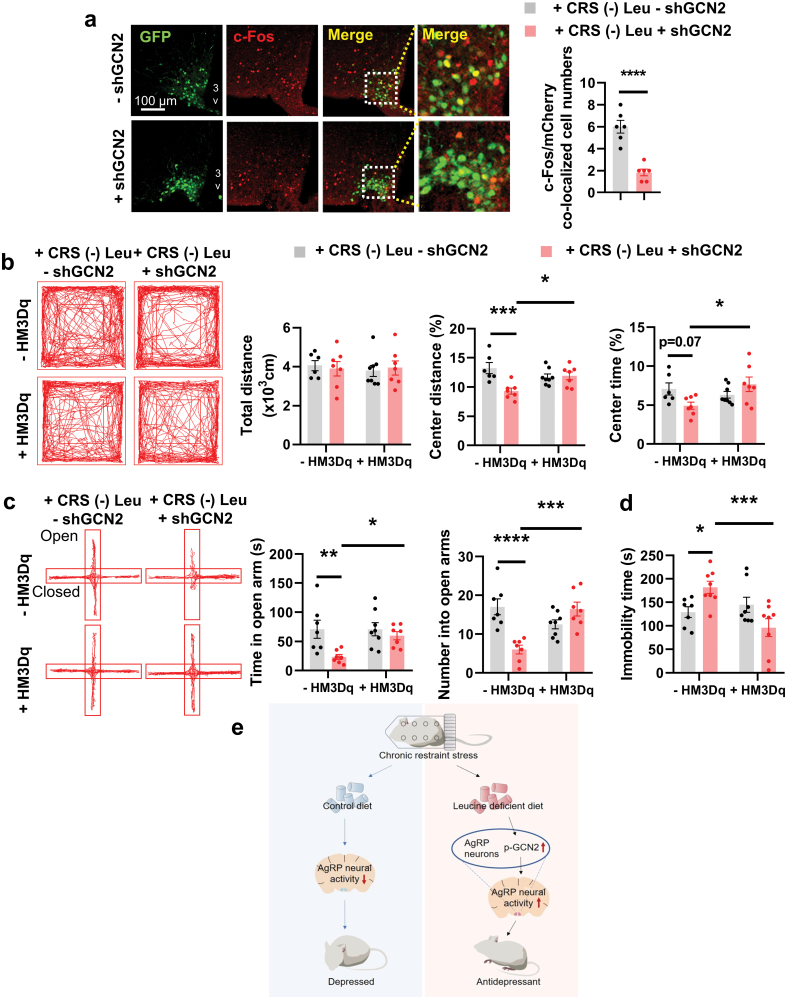
Activation of AgRP neurons reverses the effects of GCN2 knockdown on depressive behaviors. (a) IF staining for GFP (green), c-Fos (red), and merge (yellow) in the ARC (left), and quantification of c-Fos and GFP colocalized cell numbers (right); 3V, third ventricle. (b) Representative tracks of mice in the OFT, travel distance, percentage of distance in center area, and percentage of time spent in center. (c) Representative tracks of mice in the EPM, the number of entries into the open arms, and the time spent in the open arms. (d) Immobility time in the TST. (e) Summary diagram illustrating that deprivation of the EAA leucine exhibits antidepressant effects under chronic stress by stimulating GCN2 and neural activity in AgRP neurons. Studies for a were conducted using 8–9-week-old female AgRP-Cre mice receiving AAV expressing GFP (− shGCN2) or shGCN2 (+ shGCN2) with CRS fed a (-) Leu diet for 4–7 days; studies for b–d were conducted using 25–30-week-old male AgRP-Cre mice receiving AAVs expressing GFP (− shGCN2) or shGCN2 (+ shGCN2) and AAVs expressing mCherry (− hM3Dq) or hM3Dq (+ hM3Dq) with CRS fed a (-) Leu diet for 4–7 days. Data are expressed as the mean ± SEM (*n* = 6–11 per group, as indicated), with individual data points. Data were analyzed via the two-tailed unpaired Student’s *t*-test for a, or via two-way ANOVA for b–d. *P < 0.05, **P < 0.01, ***P < 0.001, ****P < 0.0001.

After confirming the role of GCN2 in AgRP neurons in leucine deficiency-induced antidepressant effects under CRS, we speculated that the blocking effect of GCN2 might be a result of the inhibited neuronal activity. We assessed AgRP neuronal activity, and found that the colocalization of GFP (reflecting AgRP neurons) and c-Fos in AgRP-shGCN2 mice was significantly lower than that in control mice (Fig. 6a). We then tested whether activation of AgRP neurons by an excitatory hM3Dq DREADD activated by CNO would reverse the blocking effect of GCN2 knockdown on leucine deprivation-induced antidepressant effects. To this end, Cre-dependent-AAVs encoding hM3Dq (AAV-DIO-hM3Dq-mCherry) or mCherry (AAV-DIO-mCherry) were injected into the ARC of AgRP-Cre mice in which GCN2 was knocked down in AgRP neurons (Supplementary Fig. S14a and b). All the mice were then subjected to CRS and given a leucine-deficient diet, with i.p. injection of CNO, 4 weeks after AAV delivery. The efficiency of activating AgRP neurons and inhibiting GCN2 in AgRP neurons by AAVs was validated by IF staining of c-Fos in AgRP neurons and examining *Gcn2* mRNA expression in the ARC (Supplementary Fig. S14c and d). Activation of AgRP neurons increased food intake, without acutely influencing body weight (Supplementary Fig. S14e−l). As expected, activation of AgRP neurons reversed the blocking effect of GCN2 knockdown in AgRP neurons on leucine deprivation-induced antidepressant effects, as reflected by the OFT, EPM, and TST (Fig. 6b−d). Similar blocking effects of GCN2 knockdown and reversal effects of AgRP neuron activation could be found in female mice, as reflected by the OFT, EPM, and TST (Supplementary Fig. S14m−q). These results suggest that GCN2 functions via activation of AgRP neurons for leucine deprivation’s protective effects.

## Discussion

The supplementation of a number of EAAs is considered helpful in improving depressive behaviors [43, 44]. However, studies on EAA deficiency in the field of depression are limited. While the depletion of several AAs, such as tryptophan and tyrosine, has been reported to aggravate depression [45, 46], whether single AA deprivation can cause or relieve depression-like behaviors remains unclear. In the current study, we surprisingly found that leucine deprivation not only has no effect on the depressive behaviors under normal conditions, but it could protect against CRS-induced depression-like behaviors. The beneficial effects observed are independent of feeding effects based on our pair-feeding experiments. Furthermore, deficiencies of other EAAs exhibit similar antidepressant properties. We then determined that these beneficial effects are dependent on AgRP neurons activated by the AA sensor GCN2 in those neurons. Our results demonstrate a novel role for deprivation of an EAA in protecting from depression induced by CRS. As epidemiological evidence strongly supports the association between depression and obesity [47], and various antidepressant drugs cause hyperphagia and obesity [4, 48], using energy restriction to alleviate depression is particularly appropriate. Though chronic leucine deprivation could cause malnutrition, our results suggest that acute deprivation or restriction of an EAA, such as leucine, may be an attractive dietary intervention, especially as it is confirmed to induce weight loss and improve glycolipid metabolism [17, 28, 29], in parallel to the relief of CRS-induced depressive behaviors.

During intake of an AA-deficient diet, various organs and tissues sense AA deficiency. We noticed that central leucine is crucial for the effect of leucine deprivation, suggesting that the hypothalamus is involved in the regulation. Interestingly, AgRP neural activity is increased after leucine deficiency both with CRS and without CRS. This finding could be partly supported by a previous study that showed that AAs inhibit *Agrp* expression in GT1-7 cells [49]. The role of AgRP neurons in the beneficial effect of leucine deprivation on depressive behaviors was demonstrated by chemogenetic intervention of AgRP neuron activity. Though hypothalamic leucine and AgRP are important for depressive behaviors, ICV injection of leucine in mice fed a leucine replete diet had no effect on the related behaviors, which may be due to the already inhibited AgRP neurons activity. Furthermore, though leucine deficiency activates AgRP neurons, it has no effects on anxiety-related behaviors in unstressed mice. Thus, in that case, it is possible that the degree of stimulation in AgRP neurons may not be enough to change anxiety-related behaviors in unstressed mice. Similar phenomena have been reported in some studies [50–52].

Another interesting finding is that AgRP neurons are activated by leucine deprivation, but the food intake is reduced. We speculate that the activated AgRP neurons could be the compensatory response to non-specific feeding inhibition; however, the stimulating effect on food intake is overcome by other inhibitory signals, such as α-MSH and signals beyond the hypothalamus [53, 54]. Despite these facts, these results suggest an important role of AgRP neurons in the protective effects of leucine deprivation against CRS-induced depression. These results also provide novel insight for the strategy for stimulating AgRP neurons, which has been shown to play many critical functions in addition to food intake [50]. For example, it has been shown that suppressed AgRP neuron activity contributes to chronic unpredictable stress (CUMS)- and high-fat-diet (HFD)-induced depression-related behaviors [24, 27]. Because leucine deprivation-induced antidepressant effects under CRS depended on AgRP neural activity, we suspect that leucine deprivation may help prevent depression-like behaviors under CUMS and HFD. Dietary restriction and fasting exerts antidepressant effects in animals, but the mechanism for these effects are currently unclear [55], and our results may help explain such beneficial effects.

It is well known that GCN2 is activated during AA deficiency [15]. We demonstrated that the leucine deprivation-induced antidepressant effects depend on GCN2 signaling. The role of GCN2 in depression remains unclear. Some studies have shown that acting downstream of GCN2, phosphorylation of α subunit of eukaryotic initiation factor 2 (p-eIF2α) contributes to Alzheimer’s disease-related memory impairments [56]. However, there have been discrepancies regarding the effects of GCN2 deletion, which may be caused by differences in the experimental conditions and animal models employed [57]. Activating transcription factor 4 (ATF4), a transcription factor acting downstream of GCN2, has important roles in synaptic plasticity and memory [58], both of which are implicated in depression development [59–61]. However, there are no direct effects of GCN2-eIF2α-ATF4 in the regulation of depression-like behaviors. We determined that GCN2 is necessary for the antidepressant effects of leucine deprivation. Since all EAA deprivation has antidepressant effects, we confirmed an important role of GCN2 in this regulation. Further, GCN2 in AgRP neurons could alter the activity of AgRP neurons and the related behaviors. However, how GCN2 regulates AgRP neuronal activity remains unknown. GCN2 also activates ATF4, which is reported to promote AgRP expression and AgRP neural activity [62]. In addition, though GCN2 is also expressed in other neurons and tissues, we did not examine GCN2 levels in other tissues or neurons, and thus the possible involvement of GCN2 in other organs or neuronal populations cannot be excluded. For instance, POMC neurons are also located in the ARC and have important roles in depressive behaviors [26]; however, the GCN2 signal in POMC neurons has not been investigated. These possibilities need to be studied in the future. Though the mechanisms underlying GCN2-mediated regulation of AgRP neuron activity are unknown, we show here for the first time that GCN2 is involved in depression, and thus it might be a potential drug target to treat this condition.

There are several questions that remain to be investigated in the future. In addition to hypothalamic AgRP neurons, other signals, neurons, or organs may be involved in the beneficial effect of leucine deprivation. For example, the HPA axis is closely related to stress and AgRP neurons [33–35]; however, the parameters of the HPA axis examined were unchanged after CRS in our studies. This might be due to the fact that the activity of the HPA axis is influenced by many factors, including the rodent strain used, the duration of the CRS, and the timing of the testing after stress [63, 64]. Recently, BCAA reduction is reported to have antidepressant effects under a HFD, through the influence on tryptophan availability in 5-HT neurons [65], suggesting another interesting possibility. In addition, previous studies showed that cortical neurons may also sense AAs and alter feeding [14], and the gut can detect macronutrients, modulating the activity of AgRP neurons via spinal afferent or vagal signaling [19, 66, 67]. Their possible involvement in our findings here requires further study.

In addition, though some studies report that ICV injection of leucine or leucinol acutely changes feeding and/or body weight [29, 36], we observed no effect on feeding or body weight after CRS upon ICV injection of either molecule, possibly due to differences in experimental design, including treatment and detection timing.

Further, though we exclude a feeding effect on the beneficial effects of leucine deprivation on CRS-induced depressive behaviors, we can not exclude the effect of body weight. As studies have found that HFD-induced body weight gain or chronic stress-induced body weight loss had no impact on anhedonia-like behavior [68, 69], we suspect that the leucine deprivation-induced weight loss may not be causally related to the depressive states.

Furthermore, we were surprised to observe that depletion of other EAAs, especially of tryptophan, also exerts antidepressant effects under CRS. Deficiency of some AAs, such as tryptophan and tyrosine, is considered to worsen depression in general [70–72]. However, the effect of EAA deficiency is not always consistent, as reported in some other studies which showed that tryptophan or tyrosine depletion does not cause depressive behaviors or even helps to recover depression [73, 74]. Furthermore, the mechanisms underlying the deficiency of AA on depressive behaviors are also different. For example, tryptophan or tyrosine deficiency induces depressive effects by decreasing their metabolites, serotonin and catecholamine, which are neurotransmitters with antidepressant effects [71, 72]. Reduced tryptophan may relieve depression-like behaviors via lowering kynurenine levels, another tryptophan metabolite leading to depression [75]. The mechanism underlying the effects of tryptophan depletion may also be mediated via activation of AgRP neurons, as we observed that AgRP neurons are stimulated under tryptophan deprivation after CRS. Therefore, the effect of individual AAs and their depletion on depression, as well as the underlying mechanisms, could be quite complex under different treatments and requires further study.

Interestingly, a recent study revealed that leucine supplementation prevents lipopolysaccharide (LPS)-induced depression [76]. However, the models for inducing depression and the underlying mechanisms are different between that study [76] and our current study. In their study, depression is induced via acute LPS administration, and leucine functions by competing with kynurenine for blood-to-brain transport [76]. In our study, depression is induced via CRS, which may not change kynurenine levels, and the effect of leucine deprivation is mediated by activating GCN2 signaling in AgRP neurons. Hence, leucine supplementation and deficiency may both exert antidepressant effects via distinct mechanisms under different models. Similarly, both supplementation and deficiency of leucine could improve glucose metabolism and induce weight loss [28, 29, 77, 78].

In conclusion, we found that deprivation of single EAAs, including leucine, has a significant protective effect on CRS-induced depression-like behaviors in mice, and these beneficial effects depend on GCN2 and AgRP neural activity (Fig. 6e). Our findings provide evidence for the efficacy of a new dietary intervention to relieve depression and establish an important role for GCN2 in depression-related behaviors, in addition to highlighting the role of AgRP neurons in AA sensing.

## Materials and methods

### Animals

All mice were of the C57BL/6J background. AgRP-Cre mice, Ai9 (tdTomato) reporter mice, and global GCN2 knockout mice (GCN2^−/−^) were obtained from the Jackson Laboratory (Bar Harbor, ME, USA). Food intake and body weight were recorded daily. Mice were maintained under a 12 h light/12 h dark cycle (lights on at 07:00 a.m./lights off at 07:00 p.m.) and 22°C–25°C, with *ad libitum* access to water and rodent standard chow diet prior to the experiments.

### Diets

Experimental Control (including all AAs), (-) Leu (leucine-deficient), (-) Ile (isoleucine-deficient), (-) Val (valine-deficient), (-) Lys (lysine-deficient), (-) Met (methionine-deficient), (-) Phe (phenylalanine-deficient) and (-) Thr (threonine-deficient), and (-) Trp (tryptophan-deficient) diets were obtained from Trophic Animal Feed High-Tech Co., Ltd (Nantong, China). Diet formulations are described in Supplementary Table 1. All diets were isocaloric and compositionally identical in terms of lipid content, with the calorie reduction in the absence of the AA compensated with carbohydrates. At the start of the feeding experiment, mice were acclimated to the control diet for 3 days and then fed with the indicated diets.

### Stereotaxic surgery and viral injections

Stereotaxic surgery was performed using a stereotaxic frame (Steolting, IL, USA) [79]. Mice body temperature was maintained using a heating pad. Ophthalmic ointment was applied to maintain eye lubrication. Viruses were injected at a rate of 50 nL/min using a micro syringe pump connected to glass pipettes. Viruses were injected into the ARC (coordinates: ML ± 0.3 mm, AP −1.5 mm, DV −5.8 mm from bregma). After injection, the glass pipettes were left in place for 8 min before withdrawal to allow for diffusion. The mice were allowed to recover from anesthesia on a heat blanket and were then i.p. injected with antibiotics (ceftriaxone sodium, 0.1 g/kg) for 3 days to prevent infection. Mice were individually housed and allowed to recover for 3–4 weeks after the surgery.

To knock down GCN2 in AgRP neurons, AgRP-Cre mice were bilaterally injected with 200 nL of Cre-dependent adeno-associated virus (AAV) vector, containing the mir-30-shGCN2 coding sequence and GFP protein in the opposite orientation flanked by two inverted loxP sites (AAV2/9-CMV-bGiobin-FLEX -mir-30-shGCN2-GFP; 4.9 × 10^12^ Pfu/mL), into the ARC [53]. Alternatively, mice were injected with an AAV vector, containing the mir-30-scramble and GFP protein in the opposite orientation flanked by two inverted loxP sites (AAV2/9-CMV-bGiobin-FLEX -mir-30-scramble-GFP; dilute to 4.9 × 10^12^ Pfu/mL), as a control. The target sequence 5ʹ-TCTGGATGGATTAGCTTATA-3ʹ for GCN2 was previously validated [36]. The AAVs would express the target sequence and GFP only in AgRP neurons 2–3 weeks after AAV delivery, by Cre-Flex system.

### ICV cannulation and drug infusion

For the ICV cannulation, a cannula was placed into the third ventricle (coordinates: ML 0 mm, AP −1.5 mm, DP −5.3 mm), and two screws were placed at the lambdoid structure to aid in supporting the cannula in the skull with dental cement. After recovery, mice were infused with 1 μL leucine (1.1 μg/μL), leucinol (10 mmol/L) or saline, and experiments were conducted 30 min later.

### DREADDs

To inhibit AgRP neural activity using DREADDs, AgRP-Cre mice were stereotaxically injected with a Cre-dependent AAV encoding an inhibitory DREADD GPCR (hM4Di) (AAV2/9-Syn-DIO-hM4Di-mCherry, 3.1 × 10^12^ Pfu/mL) or only encoding mCherry (AAV2/9-Syn-DIO-mCherry, 3.1 × 10^12^ Pfu/mL) as a control, bilaterally into the ARC at a volume of 300 nL. Four weeks after AAV delivery, all mice received i.p. injections of CNO (MedChemExpress, NJ, USA) at 1 mg/kg of body weight for hM4Di silencing [24].

To assess whether AgRP neuronal activation could reverse the blocking effect of GCN2 knockdown on leucine deprivation-induced antidepressant effects, we injected the ARC of AgRP-Cre mice with Cre-dependent AAVs expressing shGCN2 (AAV2/9-CMV-bGiobin-FLEX-mir-30-shGCN2-GFP), excitatory DREADD GPCR (hM3Dq) (AAV2/9-EF1a-DIO-hM3Dq-mCherry), and control AVVs expressing GFP (AAV2/9-CMV-bGiobin-FLEX-mir-30-scramble-GFP) or mCherry (AAV2/9-EF1a-DIO-mCherry) as indicated. Thereafter, mice were fed a leucine-deficient diet. The above AAVs were all diluted to a concentration of 3 × 10^12^ Pfu/mL, and the two indicated AAVs were mixed at a 1:1 ratio to yield a total volume of 300 nL. Four weeks after AAV delivery, all mice received i.p. injections of CNO at 0.3 mg/kg of body weight for hM3Dq activation [27].

### CRS protocol

The experiments were performed after acclimatizing the mice to the experimental environment for a week. Male WT mice (8–11 weeks old) were divided into three weight-matched groups: control group, CRS group, and CRS with leucine-deficient diet group [CRS (-) Leu] (Fig. 2). CRS and CRS (-) Leu mice were exposed to 3-h restraint (13:00–16:00) daily for 21 days in a 50 mL tube with holes that permits breathing while restricting limb movement [31]. Mice had no access to food and water during restraint. Control mice were placed in the home cage at the same time without food or water. After restraint, the mice were returned to the home cage and given food and water *ad libitum*. On CRS day 16, all the mice were acclimated to control diets for 3 days. On CRS day 19, the mice in the CRS with leucine-deficient diet group were switched to a leucine-deficient diet. Before subsequent experiments, mice were allowed to rest 1 day after CRS to exclude the effects of acute stress [80]. For mice with DREADDs, the mice were injected with CNO 30 min before the behavioral tests [27].

### Behavioral assays

All behavioral tests were performed during the afternoon. All animals were brought into the experimental room 1 h before the start of behavioral tests and remained in the same room throughout the test.

### OFT

Mice were placed in the center of a white plastic open field arena (50 cm × 50 cm × 50 cm) and allowed to explore freely for 10 min [2]. A video camera positioned directly above the arena was used to track animal movements, recorded on a computer with LabState (AniLab) to determine the total distance and the amount of time spent in the center of the chamber compared to the edges. The OFT is commonly used for assessing exploratory behavior and general activity of animals. The area was cleaned with 75% ethanol after each test to remove olfactory cues.

### EPM

The EPM consisted of a central platform (5 × 5 cm^2^), two closed arms with walls, and two opposing open arms without walls (25 cm × 5 cm). The maze was placed 60 cm above the floor. A mouse was placed in the central platform facing an open arm and was allowed to explore the maze for 5 min [2]. The time spent in the open arms and the number of entries into the open arms, were analyzed using LabState (AniLab). The area was cleaned between tests using 75% ethanol.

### TST

Mice were individually suspended about 50 cm above the surface of a table using adhesive tape that was placed roughly 1 cm from the tip of the tail. Each mouse was tested only once for 6 min [81]. The test was videotaped from the side, and the immobility time of the animal was measured in the last 5 min. Mice were considered immobile without initiated movements, and immobility was considered to include passive swaying. Video tracking data were analyzed using LabState (AniLab) software to extract the immobility time.

### FST

Mice were subjected to FST for 6 min. The FST was consisted of a cylindrical container (15 cm diameter, 25 cm height) that was filled with water (15–18 cm depth) at a temperature of 23 ± 1°C as previously described [82]. Immobility was defined as time when mice remained floating or motionless with only movements necessary for keeping balance in the water. The results are expressed as the amount of time (in seconds) that the mice spent immobile during the last 5-min of the test.

### IF staining

Mice were transcardially perfused with saline followed by PBS buffer containing 4% paraformaldehyde (PFA). Mice brains were dissected and fixed overnight at 4°C in 4% PFA, followed by cryoprotection in PBS containing 20% and 30% sucrose at 4°C. Free-floating sections (25 μm) were prepared with a cryostat. Slices were blocked for 1 h at room temperature in PBST (0.3% Triton X-100) with 5% normal donkey serum, followed by incubation with primary antibodies at 4°C overnight and secondary antibodies at room temperature for 2 h. Primary antibodies used in IF experiments included anti-GCN2 (1:500, ABclonal, Wuhan, China), anti-p-GCN2 (1:300, Biorbyt, Cambridge, UK), anti-c-Fos (1:1000, Cell Signaling Technology, MA, USA), anti-FosB (1:500, Santa Cruz, CA, USA), and anti-p-ERK (1:1000, Cell Signaling Technology, MA, USA).

### ELISA

ACTH levels in the serum of mice were measured using Mouse ACTH ELISA Kit (E-EL-M0079c, Elabscience, Wuhan, China) according to the manufacturer’s recommendations. Corticosterone levels in the serum of mice were measured using Corticosterone ELISA kits (ADI-900-097, ENZO Life Sciences, NY, USA).

### RNA isolation and RT-PCR

RNA was extracted using TRIzol reagent (Invitrogen, CA, USA). mRNA was reverse-transcribed using a High-Capacity cDNA Reverse Transcription Kit (Thermo Scientific, CA, USA) and subjected to quantitative real-time PCR analysis using SYBR Green I Master Mix reagent on an ABI 7900 system (Applied Biosystems, CA, USA). The primer sequences used in this study are described in Supplementary Table 2.

### Western blot analysis

Tissues were homogenized in ice-cold lysis buffer (50 mmol/L Tris HCl, pH 7.5, 0.5% Nonidet P-40, 150 mmol/L NaCl, 2 mmol/L EGTA, 1 mmol/L Na_3_VO_4_, 100 mmol/L NaF, 10 mmol/L Na_4_P_2_O_7_, 1 mmol/L phenylmethylsulfonyl fluoride, 10 μg/mL aprotinin, 10 μg/mL leupeptin). Tissue extracts were then immunoblotted with anti-GCN2 (1:500, ABclonal, Wuhan, China) and anti-β-actin (1:5000, Sigma, MO, USA) primary antibodies.

### Statistical analyses

Statistical analyses were performed using GraphPad Prism, version 8.0 (GraphPad Software, San Diego, CA, USA). All values are presented as the mean ± standard error of the mean (SEM). Two groups were compared using a two-tailed unpaired Student’s *t*-test. For experiments involving multiple comparisons, data were analyzed via one-way analysis of variance (ANOVA) followed by the Student–Newman–Keuls (SNK) test. The individual data points on every histogram were shown to reflect the individual variability of measures. Statistical significance was defined as *P < 0.05, **P < 0.01, ***P < 0.001, ****P < 0.0001. The sample sizes and *P* values can be found in figure legends.

## Supplementary Material

load004_suppl_Supplementary_Material

## Data Availability

All study data are included in the article and/or supporting information.

## References

[1] Malhi GS, Mann JJ. Depression. Lancet 2018; 392:2299–312. 30396512 10.1016/S0140-6736(18)31948-2

[2] Fan KQ, Li YY, Wang HL et al. Stress-induced metabolic disorder in peripheral CD4^+^ T cells leads to anxiety-like behavior. Cell 2019;179:864–79.e19. 31675497 10.1016/j.cell.2019.10.001

[3] Otte C, Gold SM, Penninx BW et al. Major depressive disorder. Nat Rev Dis Primers 2016;2:16065. 27629598 10.1038/nrdp.2016.65

[4] Sabella D. Antidepressant medications. Am J Nurs 2018;118:52–9. 10.1097/01.NAJ.0000544978.56301.f6 30138204

[5] Wang SM, Han C, Bahk WM et al. Addressing the side effects of contemporary antidepressant drugs: a comprehensive review. Chonnam Med J 2018;54:101–12. 29854675 10.4068/cmj.2018.54.2.101 PMC5972123

[6] Sarris J, Logan AC, Akbaraly TN et al. Nutritional medicine as mainstream in psychiatry. Lancet Psychiatry 2015;2:271–4. 26359904 10.1016/S2215-0366(14)00051-0

[7] Lang UE, Beglinger C, Schweinfurth N et al. Nutritional aspects of depression. Cell Physiol Biochem 2015;37:1029–43. 26402520 10.1159/000430229

[8] Kris-Etherton PM, Petersen KS, Hibbeln JR et al. Nutrition and behavioral health disorders: depression and anxiety. Nutr Rev 2021;79:247–60. 32447382 10.1093/nutrit/nuaa025 PMC8453603

[9] Yue M, Jiang J, Gao P et al. Oncogenic MYC activates a feedforward regulatory loop promoting essential amino acid metabolism and tumorigenesis. Cell Rep 2017;21:3819–32. 29281830 10.1016/j.celrep.2017.12.002

[10] Baranyi A, Amouzadeh-Ghadikolai O, von Lewinski D et al. Branched-chain amino acids as new biomarkers of major depression - a novel neurobiology of mood disorder. PLoS One 2016;11:e0160542. 27490818 10.1371/journal.pone.0160542 PMC4973973

[11] Pu J, Liu Y, Zhang H et al. An integrated meta-analysis of peripheral blood metabolites and biological functions in major depressive disorder. Mol Psychiatry 2021;26:4265–76. 31959849 10.1038/s41380-020-0645-4 PMC8550972

[12] Averina OV, Zorkina YA, Yunes RA et al. Bacterial metabolites of human gut microbiota correlating with depression. Int J Mol Sci 2020;21:9234. 33287416 10.3390/ijms21239234 PMC7730936

[13] Lukic I, Getselter D, Koren O et al. Role of tryptophan in microbiota-induced depressive-like behavior: evidence from tryptophan depletion study. Front Behav Neurosci 2019;13:123. 31231198 10.3389/fnbeh.2019.00123 PMC6558209

[14] Hao S, Sharp JW, Ross-Inta CM et al. Uncharged tRNA and sensing of amino acid deficiency in mammalian piriform cortex. Science 2005;307:1776–8. 15774759 10.1126/science.1104882

[15] Masson GR. Towards a model of GCN2 activation. Biochem Soc Trans 2019;47:1481–8. 31647517 10.1042/BST20190331 PMC6824675

[16] Ravindran R, Loebbermann J, Nakaya HI et al. The amino acid sensor GCN2 controls gut inflammation by inhibiting inflammasome activation. Nature 2016;531:523–7. 26982722 10.1038/nature17186 PMC4854628

[17] Guo F, Cavener DR. The GCN2 eIF2alpha kinase regulates fatty-acid homeostasis in the liver during deprivation of an essential amino acid. Cell Metab 2007;5:103–14. 17276353 10.1016/j.cmet.2007.01.001

[18] Hu X and Guo F. Amino acid sensing in metabolic homeostasis and health. Endocr Rev 2021;42:56–76. 33053153 10.1210/endrev/bnaa026

[19] Beutler LR, Chen Y, Ahn JS et al. Dynamics of gut-brain communication underlying hunger. Neuron 2017;96:461–75.e5. 29024666 10.1016/j.neuron.2017.09.043 PMC5691364

[20] Travaglio M, Ebling FJP. Role of hypothalamic tanycytes in nutrient sensing and energy balance. Proc Nutr Soc 2019;78:272–8. 30457065 10.1017/S0029665118002665 PMC6398574

[21] Morton GJ, Cummings DE, Baskin DG et al. Central nervous system control of food intake and body weight. Nature 2006;443:289–95. 16988703 10.1038/nature05026

[22] Augustine V, Lee S, Oka Y. Neural control and modulation of thirst, sodium appetite, and hunger. Cell 2020; 180: 25–32. 31923398 10.1016/j.cell.2019.11.040 PMC7406138

[23] Sohn JW, Elmquist JK, Williams KW. Neuronal circuits that regulate feeding behavior and metabolism. Trends Neurosci 2013; 36: 504–12. 23790727 10.1016/j.tins.2013.05.003 PMC3769497

[24] Xia G, Han Y, Meng F et al. Reciprocal control of obesity and anxiety-depressive disorder via a GABA and serotonin neural circuit. Mol Psychiatry 2021;26:2837–53. 33767348 10.1038/s41380-021-01053-w PMC8505263

[25] Shao J, Gao DS, Liu YH et al. Cav3.1-driven bursting firing in ventromedial hypothalamic neurons exerts dual control of anxiety-like behavior and energy expenditure. Mol Psychiatry 2022;27:2901–13. 35318460 10.1038/s41380-022-01513-x PMC9156408

[26] Qu N, He Y, Wang C et al. A POMC-originated circuit regulates stress-induced hypophagia, depression, and anhedonia. Mol Psychiatry 2020;25:1006–21. 31485012 10.1038/s41380-019-0506-1 PMC7056580

[27] Fang X, Jiang S, Wang J et al. Chronic unpredictable stress induces depression-related behaviors by suppressing AgRP neuron activity. Mol Psychiatry 2021;26:2299–315. 33432188 10.1038/s41380-020-01004-x PMC8272726

[28] Xiao F, Huang Z, Li H et al. Leucine deprivation increases hepatic insulin sensitivity via GCN2/mTOR/S6K1 and AMPK pathways. Diabetes 2011;60:746–56. 21282364 10.2337/db10-1246 PMC3046835

[29] Cheng Y, Zhang Q, Meng Q et al. Leucine deprivation stimulates fat loss via increasing CRH expression in the hypothalamus and activating the sympathetic nervous system. Mol Endocrinol 2011;25:1624–35. 21719534 10.1210/me.2011-0028 PMC3165911

[30] Yeo GSH, Chao DHM, Siegert AM et al. The melanocortin pathway and energy homeostasis: from discovery to obesity therapy. Mol Metab 2021;48:101206. 33684608 10.1016/j.molmet.2021.101206 PMC8050006

[31] Wang Y, Chen ZP, Hu H et al. Sperm microRNAs confer depression susceptibility to offspring. Sci Adv 2021;7:eabd7605. 33568480 10.1126/sciadv.abd7605 PMC7875527

[32] Oyola MG, Handa RJ. Hypothalamic-pituitary-adrenal and hypothalamic-pituitary-gonadal axes: sex differences in regulation of stress responsivity. Stress 2017;20:476–94. 28859530 10.1080/10253890.2017.1369523 PMC5815295

[33] Perry RJ, Lee S, Ma L et al. FGF1 and FGF19 reverse diabetes by suppression of the hypothalamic-pituitary-adrenal axis. Nat Commun 2015;6:6980. 25916467 10.1038/ncomms7980 PMC4413509

[34] Perry RJ, Resch JM, Douglass AM et al. Leptin’s hunger-suppressing effects are mediated by the hypothalamic-pituitary-adrenocortical axis in rodents. Proc Natl Acad Sci U S A 2019;116:13670–9. 31213533 10.1073/pnas.1901795116 PMC6613139

[35] Cleber Gama de Barcellos Filho P, Campos Zanelatto L, Amelia Aparecida Santana B et al. Effects chronic administration of corticosterone and estrogen on HPA axis activity and telomere length in brain areas of female rats. Brain Res 2021; 1750: 147152. 33049239 10.1016/j.brainres.2020.147152

[36] Maurin AC, Benani A, Lorsignol A et al. Hypothalamic eIF2alpha signaling regulates food intake. Cell Rep 2014;6:438–44. 24485657 10.1016/j.celrep.2014.01.006 PMC4876923

[37] Cai H, Haubensak W, Anthony TE et al. Central amygdala PKC-δ^+^ neurons mediate the influence of multiple anorexigenic signals. Nat Neurosci 2014;17:1240–8. 25064852 10.1038/nn.3767 PMC4146747

[38] Hoban AE, Stilling RM, Moloney G et al. The microbiome regulates amygdala-dependent fear recall. Mol Psychiatry 2018;23:1134–44. 28507320 10.1038/mp.2017.100 PMC5984090

[39] Tan CL, Cooke EK, Leib DE et al. Warm-sensitive neurons that control body temperature. Cell 2016;167:47–59.e15. 27616062 10.1016/j.cell.2016.08.028 PMC5062957

[40] Krashes MJ, Koda S, Ye C et al. Rapid, reversible activation of AgRP neurons drives feeding behavior in mice. J Clin Invest 2011;121:1424–8. 21364278 10.1172/JCI46229 PMC3069789

[41] Maurin AC, Jousse C, Averous J et al. The GCN2 kinase biases feeding behavior to maintain amino acid homeostasis in omnivores. Cell Metab 2005;1:273–7. 16054071 10.1016/j.cmet.2005.03.004

[42] Jing W, Zhang T, Liu J et al. A circuit of COCH neurons encodes social-stress-induced anxiety via MTF1 activation of Cacna1h. Cell Rep 2021;37:110177. 34965426 10.1016/j.celrep.2021.110177

[43] Hoepner CT, McIntyre RS, Papakostas GI. Impact of supplementation and nutritional interventions on pathogenic processes of mood disorders: a review of the evidence. Nutrients 2021;13:767. 33652997 10.3390/nu13030767 PMC7996954

[44] Sparling TM, Waid JL, Wendt AS et al. Depression among women of reproductive age in rural Bangladesh is linked to food security, diets and nutrition. Public Health Nutr 2020;23:660–73. 31915095 10.1017/S1368980019003495 PMC7058425

[45] Booij L, Van der Does AJ, Riedel WJ. Monoamine depletion in psychiatric and healthy populations: review. Mol Psychiatry 2003;8:951–73. 14647394 10.1038/sj.mp.4001423

[46] Parker G, Brotchie H. Mood effects of the amino acids tryptophan and tyrosine: ‘Food for Thought’ III. Acta Psychiatr Scand 2011;124:417–26. 21488845 10.1111/j.1600-0447.2011.01706.x

[47] Milaneschi Y, Simmons WK, van Rossum EFC et al. Depression and obesity: evidence of shared biological mechanisms. Mol Psychiatry 2019;24:18–33. 29453413 10.1038/s41380-018-0017-5

[48] Gill H, Gill B, El-Halabi S et al. Antidepressant medications and weight change: a narrative review. Obesity (Silver Spring) 2020;28:2064–72. 33022115 10.1002/oby.22969

[49] Morrison CD, Xi X, White CL et al. Amino acids inhibit *Agrp* gene expression via an mTOR-dependent mechanism. Am J Physiol Endocrinol Metab 2007;293:E165–71. 17374702 10.1152/ajpendo.00675.2006. PMC2596875

[50] Dietrich MO, Zimmer MR, Bober J et al. Hypothalamic Agrp neurons drive stereotypic behaviors beyond feeding. Cell 2015;160:1222–32. 25748653 10.1016/j.cell.2015.02.024 PMC4484787

[51] Li C, Hou Y, Zhang J et al. AGRP neurons modulate fasting-induced anxiolytic effects. Transl Psychiatry 2019;9:111. 30850579 10.1038/s41398-019-0438-1 PMC6408535

[52] Ma H, Li C, Wang J et al. Amygdala-hippocampal innervation modulates stress-induced depressive-like behaviors through AMPA receptors. Proc Natl Acad Sci U S A 2021;118:e2019409118. 33526688 10.1073/pnas.2019409118 PMC8017726

[53] Yuan F, Jiang H, Yin H et al. Activation of GCN2/ATF4 signals in amygdalar PKC-delta neurons promotes WAT browning under leucine deprivation. Nat Commun 2020;11:2847. 32504036 10.1038/s41467-020-16662-2 PMC7275074

[54] Gietzen DW, Aja SM. The brain’s response to an essential amino acid-deficient diet and the circuitous route to a better meal. Mol Neurobiol 2012;46:332–48. 22674217 10.1007/s12035-012-8283-8 PMC3469761

[55] Manchishi SM, Cui RJ, Zou XH et al. Effect of caloric restriction on depression. J Cell Mol Med 2018;22:2528–35. 29465826 10.1111/jcmm.13418 PMC5908110

[56] Ma T, Trinh MA, Wexler AJ et al. Suppression of eIF2α kinases alleviates Alzheimer’s disease-related plasticity and memory deficits. Nat Neurosci 2013;16:1299–305. 23933749 10.1038/nn.3486 PMC3756900

[57] Devi L, Ohno M. Deletion of the eIF2α Kinase GCN2 fails to rescue the memory decline associated with Alzheimer’s disease. PLoS One 2013;8:e77335. 24146979 10.1371/journal.pone.0077335 PMC3795630

[58] Wei N, Zhu LQ, Liu D. ATF4: a novel potential therapeutic target for Alzheimer’s diseas*e*. Mol Neurobiol 2015;52:1765–70. 25381575 10.1007/s12035-014-8970-8

[59] Pasini S, Corona C, Liu J et al. Specific downregulation of hippocampal ATF4 reveals a necessary role in synaptic plasticity and memory. Cell Rep 2015;11:183–91. 25865882 10.1016/j.celrep.2015.03.025 PMC4822418

[60] Smith SG, Haynes KA, Hegde AN. Degradation of transcriptional repressor ATF4 during long-term synaptic plasticity. Int J Mol Sci 2020;21:8543. 33198401 10.3390/ijms21228543 PMC7697267

[61] Price RB, Duman R. Neuroplasticity in cognitive and psychological mechanisms of depression: an integrative model. Mol Psychiatry 2020;25:530–43. 31801966 10.1038/s41380-019-0615-x PMC7047599

[62] Deng J, Yuan F, Guo Y et al. Deletion of ATF4 in AgRP neurons promotes fat loss mainly via increasing energy expenditure. Diabetes 2017;66:3142. 10.2337/db16-0954 27993927

[63] Jiang SZ, Eiden LE. Activation of the HPA axis and depression of feeding behavior induced by restraint stress are separately regulated by PACAPergic neurotransmission in the mouse. Stress 2016;19:374–82. 27228140 10.1080/10253890.2016.1174851 PMC5564370

[64] Algamal M, Pearson AJ, Hahn-Townsend C et al. Repeated unpredictable stress and social isolation induce chronic HPA axis dysfunction and persistent abnormal fear memory. Prog Neuropsychopharmacol Biol Psychiatry 2021;104:110035. 32682873 10.1016/j.pnpbp.2020.110035

[65] Zemdegs J, Martin H, Pintana H et al. Metformin promotes anxiolytic and antidepressant-like responses in insulin-resistant mice by decreasing circulating branched-chain amino acids. J Neurosci 2019;39:5935–48. 31160539 10.1523/JNEUROSCI.2904-18.2019 PMC6650994

[66] Goldstein N, McKnight AD, Carty JRE et al. Hypothalamic detection of macronutrients via multiple gut-brain pathways. Cell Metab 2021;33:676–87.e5. 33450178 10.1016/j.cmet.2020.12.018 PMC7933100

[67] Su Z, Alhadeff AL, Betley JN. Nutritive, post-ingestive signals are the primary regulators of AgRP neuron activity. Cell Rep 2017;21:2724–36. 29212021 10.1016/j.celrep.2017.11.036 PMC5724395

[68] Yoshizaki K, Asai M, Hara T. High-fat diet enhances working memory in the Y-maze test in male C57BL/6J mice with less anxiety in the elevated plus maze test. Nutrients 2020;12: 2036. 32659954 10.3390/nu12072036 PMC7400900

[69] Willner P, Moreau JL, Nielsen CK et al. Decreased hedonic responsiveness following chronic mild stress is not secondary to loss of body weight. Physiol Behav 1996;60:129–34. 8804652 10.1016/0031-9384(95)02256-2

[70] Kraus C, Castren E, Kasper S et al. Serotonin and neuroplasticity - links between molecular, functional and structural pathophysiology in depression. Neurosci Biobehav Rev 2017;77:317–26. 28342763 10.1016/j.neubiorev.2017.03.007

[71] Dell’Osso L, Carmassi C, Mucci F et al. Depression, serotonin and tryptophan. Curr Pharm Des 2016;22:949–54. 26654774 10.2174/1381612822666151214104826

[72] Gelenberg AJ, Gibson CJ. Tyrosine for the treatment of depression. Nutr Health 1984;3:163–73. 6443584 10.1177/026010618400300305

[73] van Donkelaar EL, Blokland A, Lieben CK et al. Acute tryptophan depletion in C57BL/6 mice does not induce central serotonin reduction or affective behavioural changes. Neurochem Int 2010;56:21–34. 19716853 10.1016/j.neuint.2009.08.010

[74] Jans LA, Riedel WJ, Markus CR et al. Serotonergic vulnerability and depression: assumptions, experimental evidence and implications. Mol Psychiatry 2007;12:522–43. 17160067 10.1038/sj.mp.4001920

[75] Marx W, McGuinness AJ, Rocks T et al. The kynurenine pathway in major depressive disorder, bipolar disorder, and schizophrenia: a meta-analysis of 101 studies. Mol Psychiatry 2021;26:4158–78. 33230205 10.1038/s41380-020-00951-9

[76] Walker AK, Wing EE, Banks WA et al. Leucine competes with kynurenine for blood-to-brain transport and prevents lipopolysaccharide-induced depression-like behavior in mice. Mol Psychiatry 2019;24:1523–32. 29988087 10.1038/s41380-018-0076-7 PMC6326900

[77] Su Y, Lam TK, He W et al. Hypothalamic leucine metabolism regulates liver glucose production. Diabetes 2012;61:85–93. 22187376 10.2337/db11-0857 PMC3237640

[78] Blouet C, Jo YH, Li X et al. Mediobasal hypothalamic leucine sensing regulates food intake through activation of a hypothalamus-brainstem circuit. J Neurosci 2009;29:8302–11. 19571121 10.1523/JNEUROSCI.1668-09.2009 PMC2740923

[79] Cetin A, Komai S, Eliava M et al. Stereotaxic gene delivery in the rodent brain. Nat Protoc 2006;1:3166–73. 17406580 10.1038/nprot.2006.450

[80] Zhu X, Tang HD, Dong WY et al. Distinct thalamocortical circuits underlie allodynia induced by tissue injury and by depression-like states. Nat Neurosci 2021;24:542–53. 33686297 10.1038/s41593-021-00811-x

[81] Zhou W, Jin Y, Meng Q et al. A neural circuit for comorbid depressive symptoms in chronic pain. Nat Neurosci 2019;22:1649–58. 31451801 10.1038/s41593-019-0468-2

[82] Yang Y, Cui Y, Sang K et al. Ketamine blocks bursting in the lateral habenula to rapidly relieve depression. Nature 2018;554:317–22. 29446381 10.1038/nature25509

