# Supplementary material for: Leucine deprivation results in antidepressant effects via GCN2 in AgRP neurons
Source: Life Metab. 2023 Feb 4;2(1):load004. doi: 10.1093/lifemeta/load004 (PMC11748975; doi:10.1093/lifemeta/load004)
Supplement: load004_suppl_Supplementary_Material [file load004_suppl_Supplementary_Material.doc]

**Supplemental information**

**Leucine deprivation results in antidepressant effects via GCN2 in AgRP neurons**

**Authors**

Feixiang Yuan^1^**^#^**, Shangming Wu^2^**^#^**, Ziheng Zhou^2^**^#^**, Fuxin Jiao^2^, Hanrui Yin^2^, Yuguo Niu^1^, Haizhou Jiang^2^, Shanghai Chen^1^ and Feifan Guo^1*^

**Affiliations**

^1^Zhongshan Hospital, State Key Laboratory of Medical Neurobiology, Institute for Translational Brain Research, MOE Frontiers Center for Brain Science, Fudan University, Shanghai, 200032, China

^2^Chinese Academy of Sciences (CAS) Key Laboratory of Nutrition, Metabolism and Food Safety, Innovation Center for Intervention of Chronic Disease and Promotion of Health, Shanghai Institute of Nutrition and Health, University of Chinese Academy of Sciences, Chinese Academy of Sciences, Shanghai, 200031, China

**^#^**These authors contributed equally to this work

*Correspondence to: Feifan Guo

Email: ffguo@fudan.edu.cn; Tel: 86-21-5423-7056; Fax: 0086 21 5423 7056

Address: 131 Dongan Road, Shanghai, China 200032

**Running title：**Leucine deprivation relieves depressive behaviors

**Supplemental Figures**


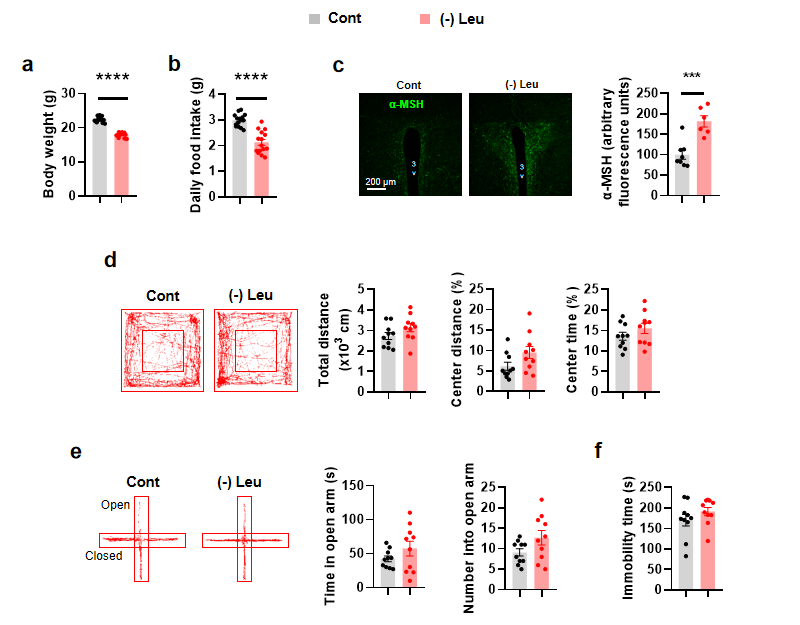


**Figure S1. Parameters of mice subjected to short-term leucine deprivation.**

(a) Body weight. (b) Average of daily food intake. (c) Immunofluorescence (IF) staining for α-MSH in hypothalamus (left), and quantification of α-MSH fluorescence (right). (d) Representative tracks of mice in OFT, travel distance, percentage of distance in center area and percentage of time spent in center. (e) Representative tracks of mice in EPM, the number of entries into the open arms and the time spent in the open arms. (f) Immobility time of TST. Studies for a and b were conducted using 8–9-week-old male wild-type mice fed a Cont or (-) Leu diet for 4-7 days; studies for c-f were conducted using 8–9-week-old female wild-type mice fed a Cont or (-) Leu diet for 4-7 days. Data are expressed as the mean ± SEM (n = 6-16 per group, as indicated), with individual data points. Data were analyzed via two-tailed unpaired Student’s *t*-test. ****P* < 0.001, *****P* < 0.0001.


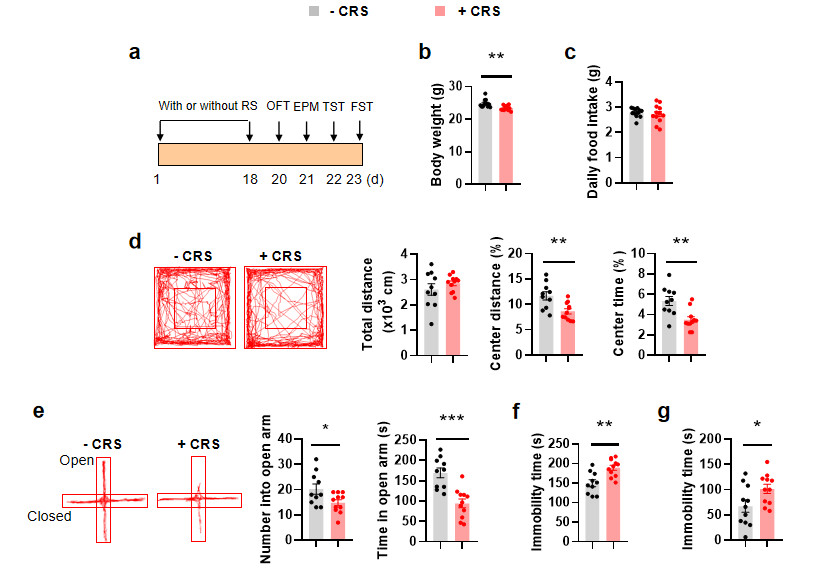


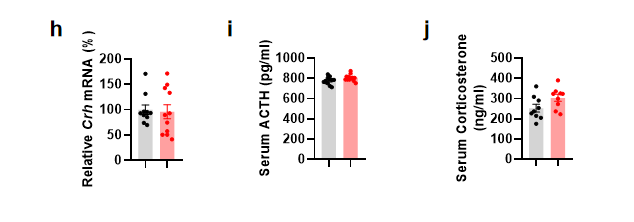


**Figure S2. Chronic restraint stress (CRS) induces depression-like behaviors.** (a) Timeline of the restraint or control protocol and behavioral tests. OFT, open field test; EPM, elevated plus maze test; TST, tail suspension test; FST, forced swim test. (b) Body weight. (c) Average of daily food intake. (d) Representative tracks of mice in the OFT, travel distance, percentage of distance in center area and percentage of time spent in center. (e) Representative tracks of mice in the EPM, the number of entries into the open arms and the time spent in the open arms. (f and g) Immobility time of TST and FST respectively. (h) Gene expression of *Crh* in hypothalamus by RT-PCR. (i) ACTH levels in serum by ELISA. (j) Corticosterone levels in serum by ELISA. Studies were conducted using 8–9-week-old male wild-type mice with or without CRS. Data are expressed as the mean ± SEM (n = 9-13 per group, as indicated), with individual data points. Data were analyzed via two-tailed unpaired Student’s *t*-test. **P* < 0.05, ***P* < 0.01, ****P* < 0.001.


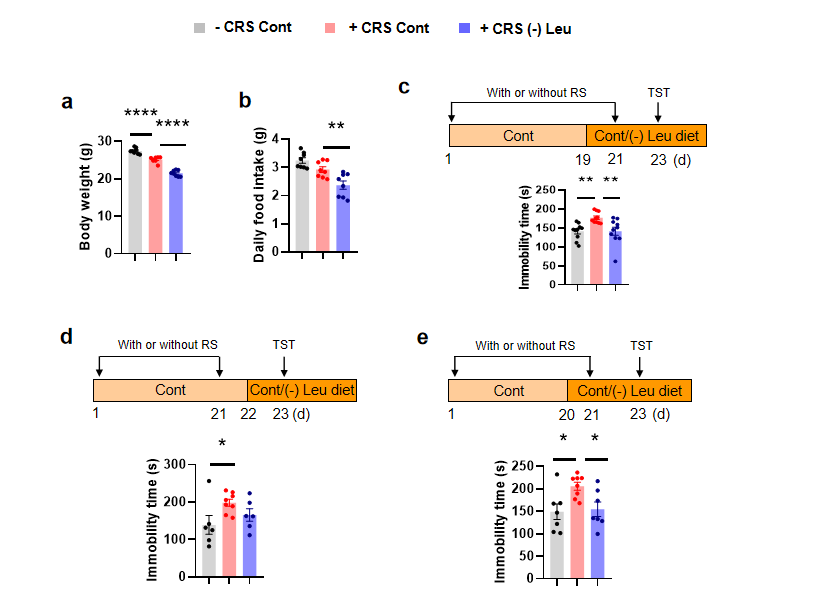


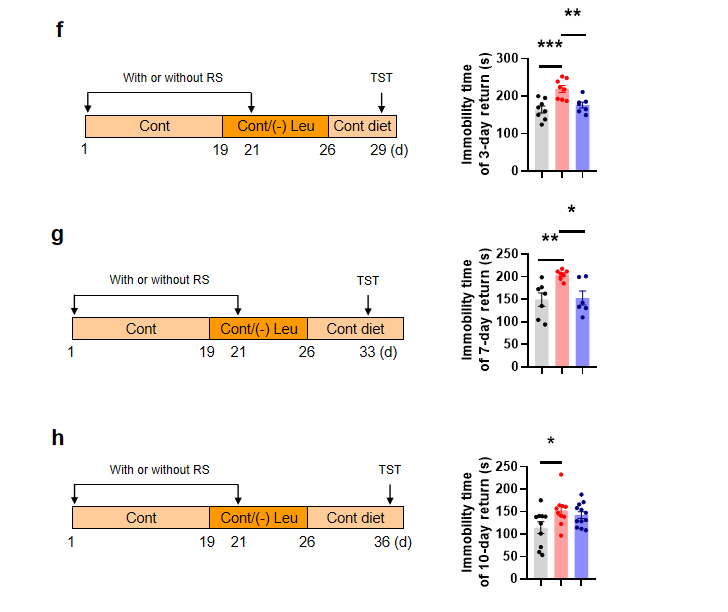


**Figure S3. Parameters of mice subjected to CRS and fed an acute leucine-deficient diet.**

(a) Body weight.

(b) Average of daily food intake.

(c-h) Schematic diagram illustrating the timeline of the restraint or control protocol and behavioral tests, and the immobility time of the tail suspension test (TST).

Studies for a and b were conducted using 8–9-week-old male wild-type (WT) mice with or without CRS fed a control (Cont) or leucine-deficient [(-) Leu] diet for 4-7 days; study for c was conducted using 8–9-week-old female WT mice with or without CRS fed a Cont or (-) Leu diet for 4-7 days; studies for d and e were conducted using 8–9-week-old male WT mice with or without CRS fed a Cont or (-) Leu diet for 1 day (d) or 3 days (e); studies for f-h were conducted using 12-14-week-old male wild-type mice with or without CRS fed a Cont or (-) Leu diet for 7 days, then fed a control diet for 3 days (f), 7 days (g) or 10 days (h). Data are expressed as the mean ± SEM (n = 6-12 per group, as indicated), with individual data points. Data were analyzed via one-way ANOVA followed by the SNK (Student–Newman–Keuls) test. **P* < 0.05, ***P* < 0.01, ****P* < 0.001, *****P* < 0.0001.


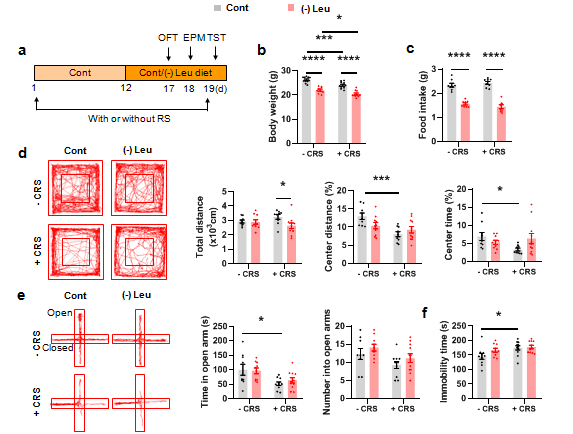


**Figure S4. Leucine deprivation during CRS does not relieve CRS-induced depressive behaviors.**

(a) Timeline of the restraint or control protocol and behavioral tests. OFT, open field test; EPM, elevated plus maze test; TST, tail suspension test.

(b) Body weight.

(c) Average of daily food intake.

(d) Representative tracks of mice in the OFT, travel distance, percentage of distance in center area and percentage of time spent in center.

(e) Representative tracks of mice in the EPM, the number of entries into the open arms and the time spent in the open arms.

(f) Immobility time of TST.

Studies were conducted using 10-12-week-old male wild-type mice with or without CRS fed a control (Cont) or leucine-deficient [(-) Leu] diet for 4-7 days. Data are expressed as the mean ± SEM (n = 8-11 per group, as indicated), with individual data points. Data were analyzed via two-way ANOVA. **P* < 0.05, ****P* < 0.001, *****P* < 0.0001.


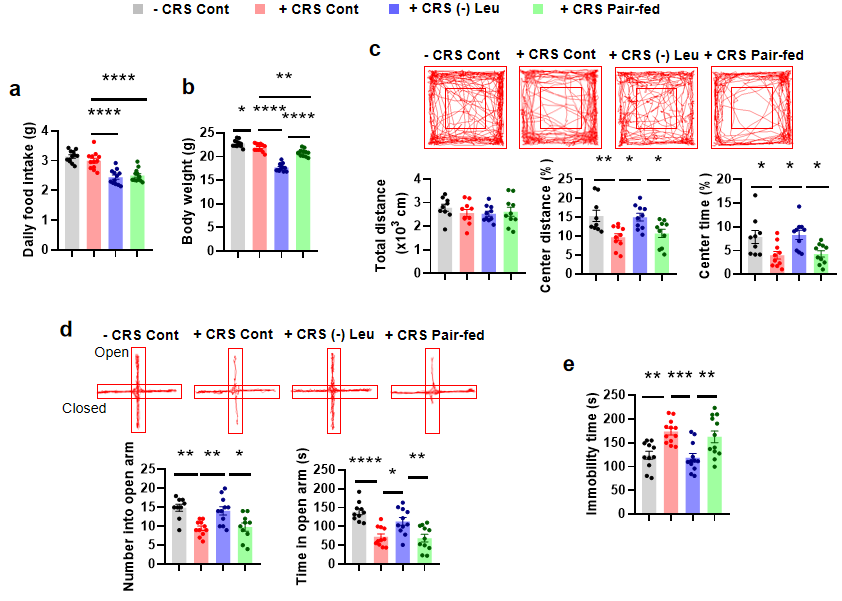


**Figure S5. Pair-feeding with a control diet to mimic the intake of a leucine-deficient diet does not influence depressive behaviors.**

(a) Average of daily food intake.

(b) Body weight.

(c) Representative tracks of mice in the open field test (OFT), travel distance, percentage of distance in center area and percentage of time spent in center.

(d) Representative tracks of mice in the elevated plus maze test (EPM), the number of entries into the open arms and the time spent in the open arms.

(e) Immobility time of the tail suspension test (TST).

Studies were conducted using 8–9-week-old male wild-type mice with or without CRS fed a control (Cont), leucine-deficient [(-) Leu], or pair-fed (Pair-fed) diet for 4-7 days. Data are expressed as the mean ± SEM (n = 9-12 per group, as indicated), with individual data points. Data were analyzed via one-way ANOVA followed by the SNK (Student–Newman–Keuls) test. **P* < 0.05, ***P* < 0.01, ****P* < 0.001, *****P* < 0.0001.


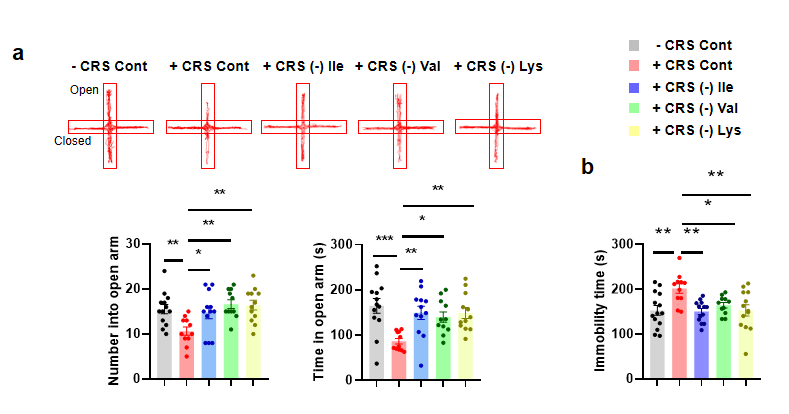


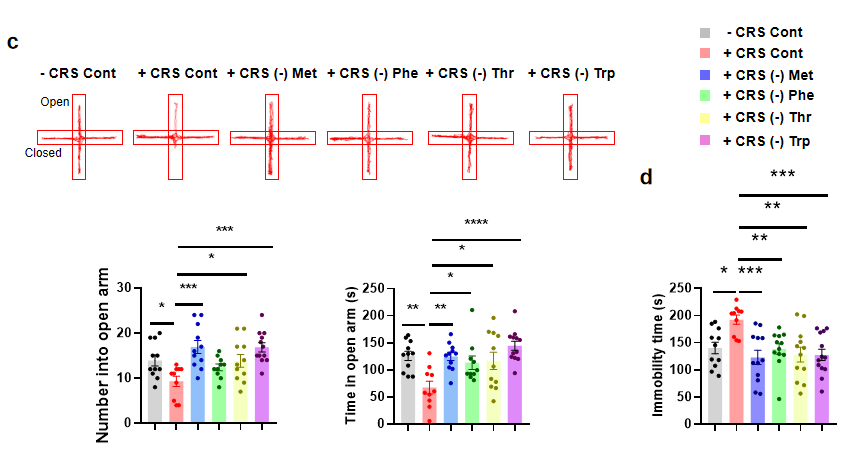


**Figure S6. Deficiency of other essential amino acids has antidepressant effects in the CRS model.**

(a and c) Representative tracks of mice in the elevated plus maze test (EPM), the number of entries into the open arms and the time spent in the open arms.

(b and d) Immobility time of the tail suspension test (TST).

Studies for a and b were conducted using 9–11-week-old male wild-type (WT) mice with or without CRS fed a control (Cont), isoleucine-deficient [(-) Ile], valine-deficient [(-) Val] and lysine-deficient [(-) Lys] diet for 4-7 days; studies for c and d were conducted using 9–11-week-old male WT mice with or without CRS fed a Cont, methionine-deficient [(-) Met], phenylalanine-deficient [(-) Phe], threonine-deficient [(-) Thr] and tryptophan-deficient [(-) Trp] diet for 4-7 days. Data are expressed as the mean ± SEM (n = 10-14 per group, as indicated), with individual data points. Data were analyzed via one-way ANOVA followed by the SNK (Student–Newman–Keuls) test. **P* < 0.05, ***P* < 0.01, ****P* < 0.001.


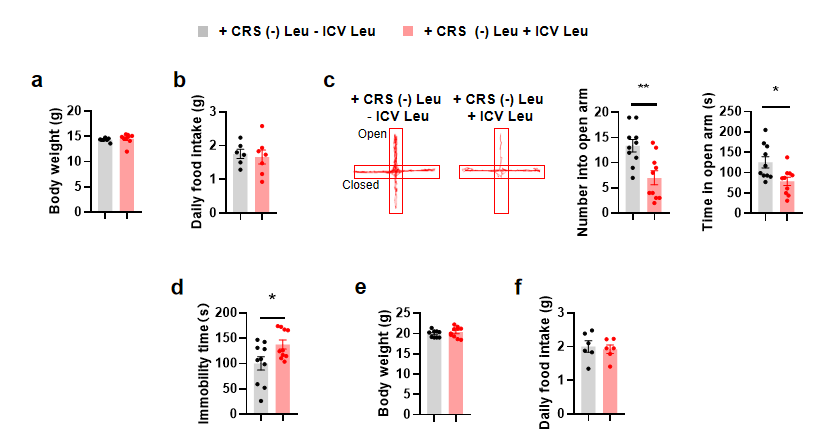


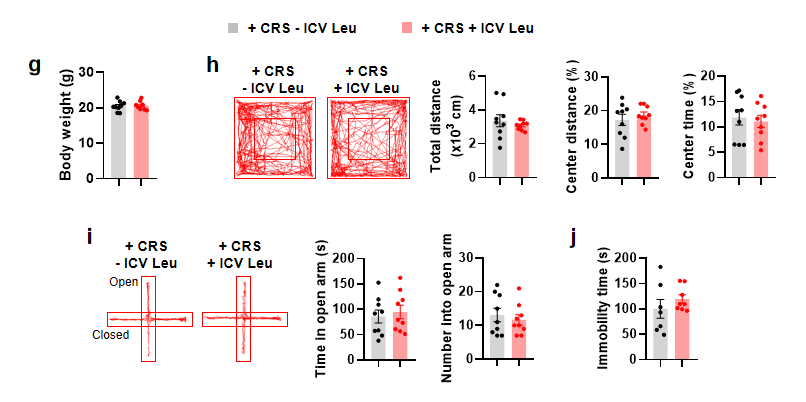


**Figure S7. Parameters of mice with intracerebroventricular (ICV) injection of leucine in the CRS model.**

(a, e and g) Body weight.

(b and f) Average of daily food intake.

(c and i) Representative tracks of mice in the elevated plus maze test (EPM), the number of entries into the open arms and the time spent in the open arms.

(d and j) Immobility time of the tail suspension test (TST).

(h) Representative tracks of mice in the open field test (OFT), travel distance, percentage of distance in center area and percentage of time spent in center.

Studies for a and b were conducted using 7–8-week-old female wild-type (WT) mice with CRS fed a leucine-deficient [(-) Leu] diet with ICV of saline (- ICV Leu) or leucine (+ ICV Leu) for 4-7 days; studies for c-f were conducted using 8-9-week-old male WT mice with CRS fed a (-) Leu diet with ICV of saline (- ICV Leu) or leucine (+ ICV Leu) for 4-7 days; studies g-j were conducted using 7-8-week-old male WT mice with CRS fed a control diet with ICV of saline (- ICV Leu) or leucine (+ ICV Leu) for 4-7 days. Data are expressed as the mean ± SEM (n = 6-10 per group, as indicated), with individual data points. Data were analyzed via two-tailed unpaired Student’s *t*-test. **P* < 0.05, ***P* < 0.01.


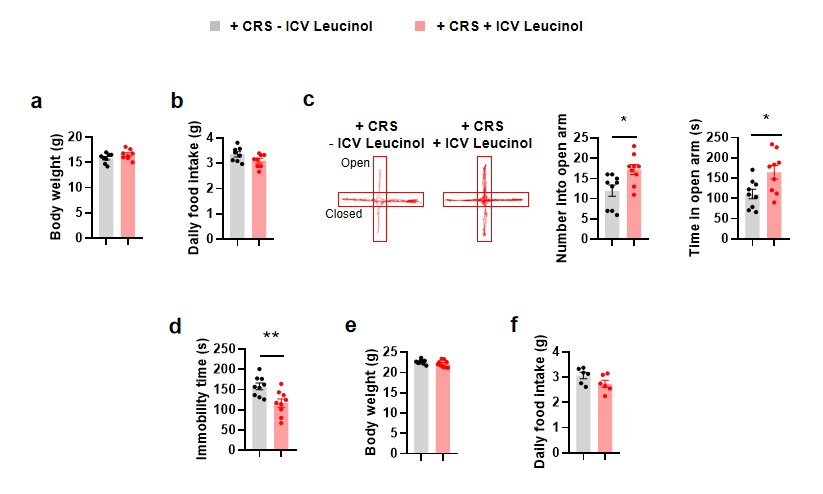


**Figure S8. Parameters of mice with intracerebroventricular (ICV) injection of leucinol in the CRS model.**

(a and e) Body weight.

(b and f) Average of daily food intake.

(c) Representative tracks of mice in the elevated plus maze test (EPM), the number of entries into the open arms and the time spent in the open arms.

(d) Immobility time of the tail suspension test (TST).

Studies for a and b were conducted using 7–8-week-old female WT mice with CRS fed a control diet with ICV of saline (- ICV Leucinol) or leucinol (+ ICV Leucinol) for 4-7 days; studies for c-f were conducted using 8-9-week-old male WT mice with CRS fed a control diet with ICV of saline (- ICV Leucinol) or leucinol (+ ICV Leucinol) for 4-7 days. Data are expressed as the mean ± SEM (n = 6-9 per group, as indicated), with individual data points. Data were analyzed via two-tailed unpaired Student’s *t*-test. **P* < 0.05, ***P* < 0.01.


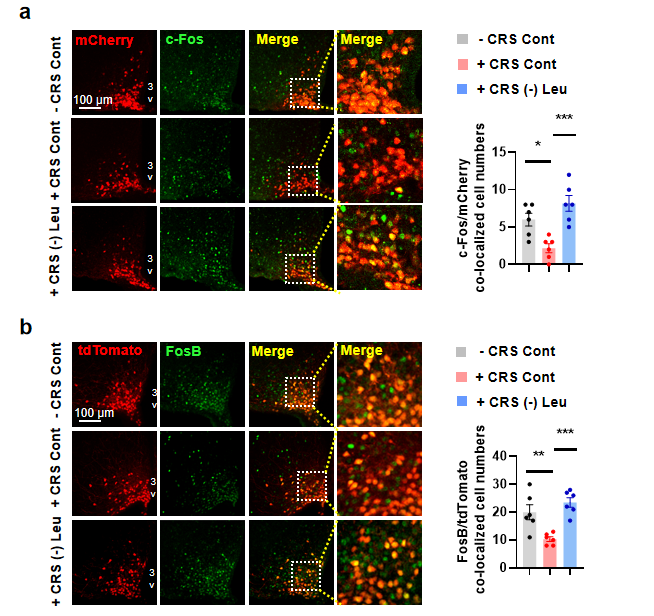


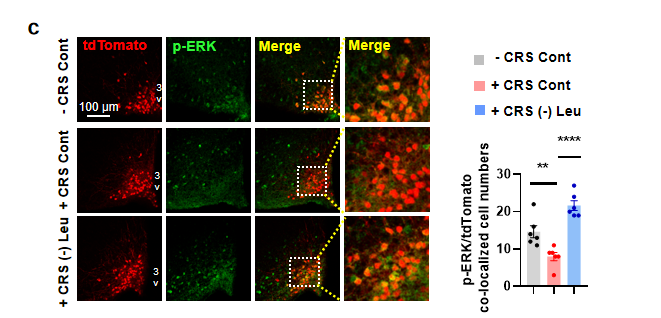


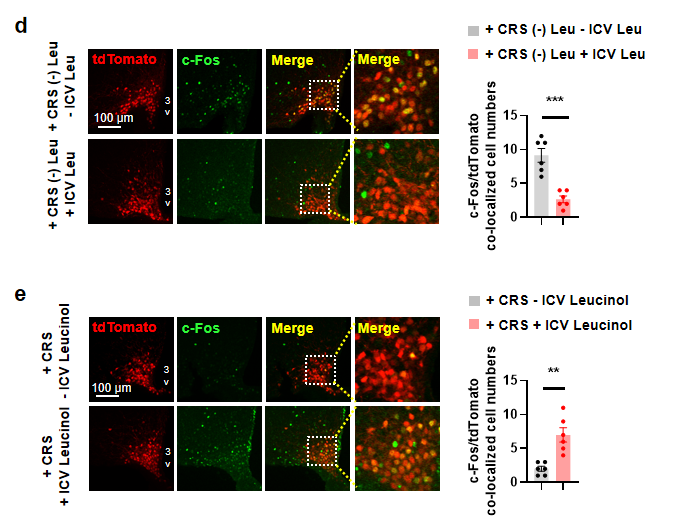


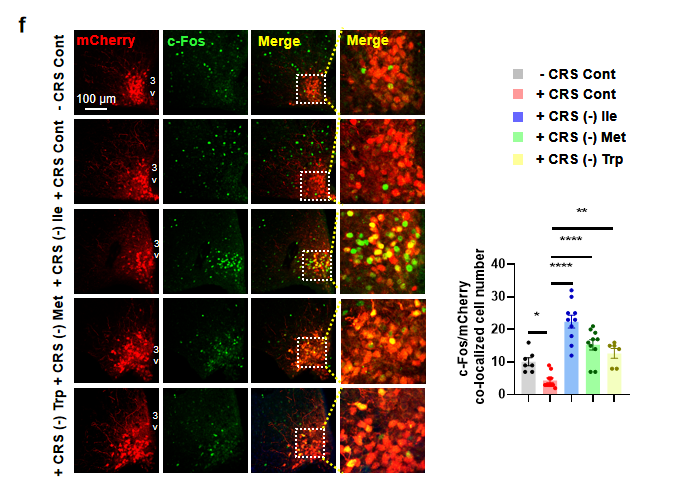


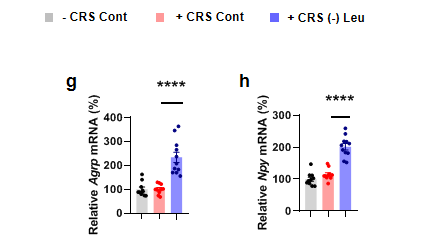


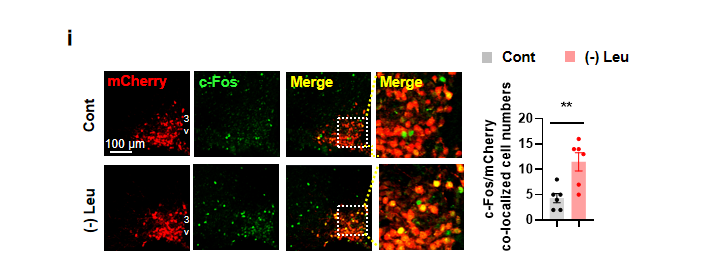


**Figure S9. Activity-related signals in AgRP neurons under different conditions.**

(a and f) Immunofluorescence (IF) staining for mCherry (red), c-Fos (green) or merge (yellow) in arcuate nucleus (ARC) (left), and quantification of c-Fos and mCherry colocalized cell numbers (right); 3V, third ventricle.

(b) IF staining for tdTomato (red), FosB (green) or merge (yellow) in ARC (left), and quantification of FosB and tdTomato colocalized cell numbers (right).

(c) IF staining for tdTomato (red), p-ERK (green) or merge (yellow) in ARC (left), and quantification of p-ERK and tdTomato colocalized cell numbers (right).

(d and e) IF staining for tdTomato (red), c-Fos (green) or merge (yellow) in ARC (left), and quantification of c-Fos and tdTomato colocalized cell numbers (right).

(g and h) Gene expression of *Agrp* and *Npy* in hypothalamus by RT-PCR.

1. IF staining for mCherry (red), c-Fos (green) or merge (yellow) in ARC (left), and quantification of c-Fos and mCherry colocalized cell numbers (right).

Studies for a were conducted using 8-9-week-old male AgRP-Cre mice receiving AAVs expressing mCherry, with or without CRS fed a control (Cont) or leucine-deficient [(-) Leu] diet for 4-7 days; studies for b and c were conducted using 8–9-week-old female AgRP-Ai9 mice with or without CRS fed a Cont or (-) Leu diet for 4-7 days; studies for d were conducted using 10–11-week-old male AgRP-Ai9 mice with CRS fed a (-) Leu diet with ICV of saline (- ICV Leu) or leucine (+ ICV Leu) for 3 days; studies for e conducted using 10–11-week-old male AgRP-Ai9 mice with CRS fed a control diet with ICV of saline (- ICV Leucinol) or leucinol (+ ICV Leucinol) for 3 days; studies for f were conducted using 9–10-week-old female AgRP-Cre mice receiving AAVs expressing mCherry into ARC, with or without CRS, fed a Cont, isoleucine-deficient [(-) Ile], methionine-deficient [(-) Met] or tryptophan-deficient [(-) Trp] diet for 5 days; studies for g and h were conducted using 8-9-week-old male wild-type mice with or without CRS fed a Cont or (-) Leu diet for 7 days; studies for i were conducted using 8-9-week-old female AgRP-Cre mice receiving AAVs expressing mCherry, fed a Cont or (-) Leu diet for 7 days without CRS. Data are expressed as the mean ± SEM (n = 6-11 per group, as indicated), with individual data points. Data were analyzed via one-way ANOVA followed by the SNK (Student–Newman–Keuls) test for a, b, c and f-h, or via two-tailed unpaired Student’s *t* test for d and e. **P* < 0.05, ***P* < 0.01, ****P* < 0.001, *****P* < 0.0001.


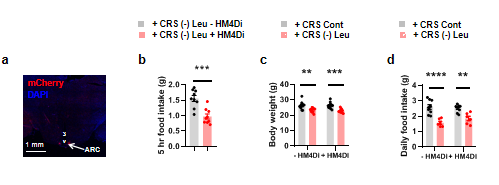


**Figure S10. Parameters of mice with inhibition of AgRP neural activity subjected to CRS and fed a leucine-deficient diet.**

(a) Post hoc visualization of mCherry (red) and DAPI (blue) in arcuate nucleus (ARC) of AgRP-Cre mice receiving AAV-DIO-HM4Di-mCherry stereotaxic injections; 3V, third ventricle.

(b) 5-hour food intake after fasting.

(c) Body weight.

(d) Average of daily food intake.

Studies were conducted using 8–16-week-old male AgRP-Cre mice receiving AAVs expressing mCherry (- HM4Di) or HM4Di (+ HM4Di) with or without CRS fed a leucine-deficient [(-) Leu] diet for 4-7 days. Data are expressed as the mean ± SEM (n = 6-11 per group, as indicated), with individual data points. Data were analyzed via two-tailed unpaired Student’s *t*-test for b, or via two-way ANOVA for c-d. ***P* < 0.01, ****P* < 0.001, *****P* < 0.0001.


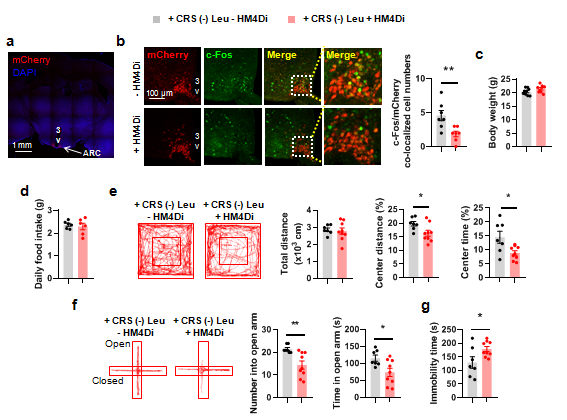


**Figure S11. Inhibition of AgRP** **neural activity blocks leucine deprivation-induced antidepressant effects in female mice.**

1. Post hoc visualization of mCherry (red) and DAPI (blue) in arcuate nucleus (ARC) of AgRP-Cre mice receiving AAV-DIO-HM4Di-mCherry stereotaxic injections; 3V, third ventricle.
2. Immunofluorescence (IF) staining for mCherry (red), c-Fos (green) or merge (yellow) in ARC (left), and quantification of c-Fos and mCherry colocalized cell numbers (right).
3. Body weight.
4. Average of daily food intake.

(e) Representative tracks of mice in the open field test (OFT), travel distance, percentage of distance in center area and percentage of time spent in center.

(f) Representative tracks of mice in the elevated plus maze test (EPM), the number of entries into the open arms and the time spent in the open arms.

(g) Immobility time of the tail suspension test (TST).

Studies were conducted using 8-9-week-old female AgRP-Cre mice receiving AAVs expressing mCherry (- HM4Di) or HM4Di (+ HM4Di) with CRS fed a leucine-deficient [(-) Leu] diet for 4-7 days. Data are expressed as the mean ± SEM (n = 6-9 per group, as indicated), with individual data points. Data were analyzed via two-tailed unpaired Student’s *t*-test. **P* < 0.05, ***P* < 0.01.


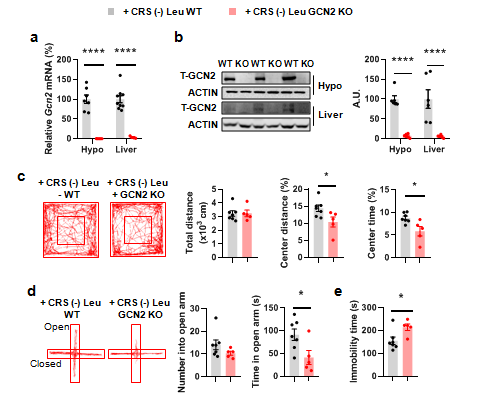


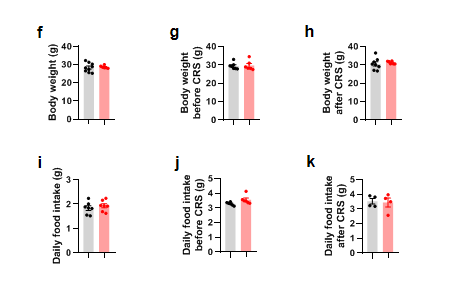


**Figure S12. Global GCN2 deletion disrupts the beneficial effects of a leucine-deficient diet on depressive behaviors.**

(a) Gene expression of *Gcn2* in hypothalamus (hypo) and liver by RT-PCR.

(b) GCN2 protein in hypothalamus and liver by western blotting (left) and quantified by densitometric analysis (right), A.U.: arbitrary units.

(c) Representative tracks of mice in the open field test (OFT), travel distance, percentage of distance in center area and percentage of time spent in center.

(d) Representative tracks of mice in the elevated plus maze test (EPM), the number of entries into the open arms and the time spent in the open arms.

(e) Immobility time of the tail suspension test (TST).

(f-h) Body weight.

(i-k) Average of daily food intake.

Studies were conducted using 12–14-week-old male wild-type (WT) or GCN2^-/-^ (GCN2 KO) mice with CRS fed a leucine-deficient [(-) Leu] diet for 4-7 days. Data are expressed as the mean ± SEM (n = 4-9 per group, as indicated), with individual data points. Data were analyzed via two-tailed unpaired Student’s *t* test. **P* < 0.05, *****P* < 0.0001.


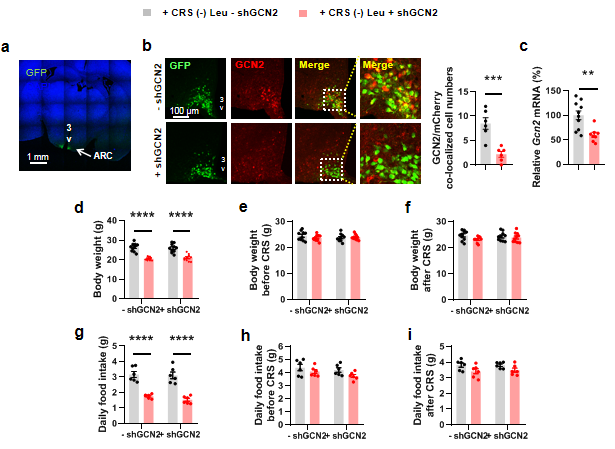


**Figure S13. Parameters of mice with GCN2 knockdown in AgRP neurons subjected to CRS and fed a leucine-deficient diet.**

(a) Post hoc visualization of GFP (green) and DAPI (blue) in arcuate nucleus (ARC) of AgRP-Cre mice receiving AAV-FLEX-shGCN2-GFP stereotaxic injections; 3V, third ventricle.

(b) Immunofluorescence (IF) staining for GFP (green), GCN2 (red) or merge (yellow) in ARC (left), and quantification of GCN2 and GFP colocalized cell numbers (right). (c) Gene expression of *Gcn2* in ARC by RT-PCR.

(d-f) Body weight.

(g-i) Average of daily food intake.

Studies were conducted using 8–9-week-old female AgRP-Cre mice receiving AAVs expressing GFP (- shGCN2) or shGCN2 (+ shGCN2) with CRS fed a leucine-deficient diet for 4-7 days. Data are expressed as the mean ± SEM (n = 6-10 per group, as indicated), with individual data points. Data were analyzed via two-tailed unpaired Student’s *t*-test for b-c, or via two-way ANOVA for d-i. ***P* < 0.01, ****P* < 0.001, *****P* < 0.0001.


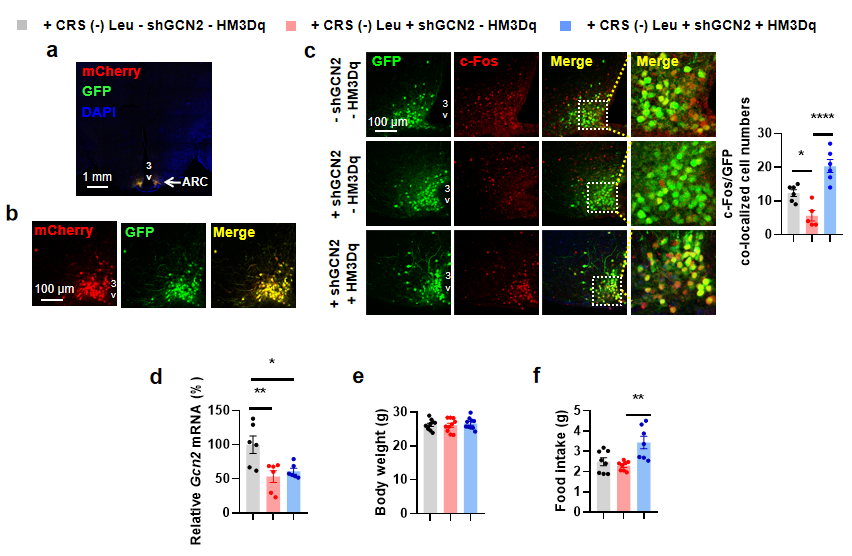


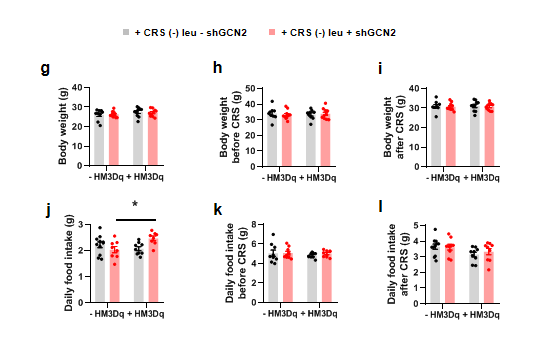


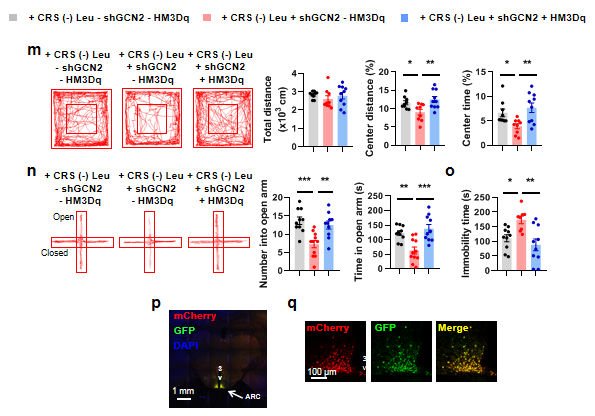


**Figure S14. Parameters of mice with GCN2 knockdown and neuron stimulation in AgRP neurons subjected to CRS and fed a leucine deficient diet.**

(a and p) Post hoc visualization of GFP (green), mCherry (red) and DAPI (blue) in arcuate nucleus (ARC) of AgRP-Cre mice receiving AAV-FLEX-shGCN2-GFP and AAV-DIO-HM3Dq-mCherry stereotaxic injections; 3V, third ventricle.

(b and q) Immunofluorescence (IF) staining for GFP (green), mCherry (red) or merge (yellow) in ARC (left).

(c) IF staining for GFP (green), c-Fos (red) or merge (yellow) in ARC (left), and quantification of c-Fos and GFP colocalized cell numbers (right).

(d) Gene expression of *Gcn2* in ARC by RT-PCR.

(e, g-i) Body weight.

(f, j-l) Average of daily food intake.

(m) Representative tracks of mice in the open field test (OFT), travel distance, percentage of distance in center area and percentage of time spent in center.

(n) Representative tracks of mice in the elevated plus maze test (EPM), the number of entries into the open arms and the time spent in the open arms.

(o) Immobility time of the tail suspension test (TST).

Studies for a-d, g-l were conducted using 10-30-week-old male AgRP-Cre mice, and studies for e, f, m-o were conducted using 8-9-week-old female AgRP-Cre mice, receiving AAVs expressing GFP (- shGCN2) or shGCN2 (+ shGCN2) and AAVs expressing mCherry (- HM3Dq) or HM3Dq (+ HM3Dq) with CRS fed a (-) Leu diet for 4-7 days. Data are expressed as the mean ± SEM (n = 5-11 per group, as indicated), with individual data points. Data were analyzed via one-way ANOVA followed by the SNK (Student-Newman-Keuls) test for c-f, m-o, or via two-way ANOVA for g-l. **P* < 0.05, ***P* < 0.01, ****P* < 0.001, *****P* < 0.0001.

**Supplementary Table. 1 Diet composition used in current study**

| Ingredient (gm） | Control diet | (-) Leu diet | (-) Ile diet | (-) Val diet | (-) Lys diet |
| --- | --- | --- | --- | --- | --- |
| L-Arginine | 10 | 10 | 10 | 10 | 10 |
| L-Histidine-HCl-H2O | 6 | 6 | 6 | 6 | 6 |
| L-Isoleucine | 8 | 8 | 0 | 8 | 8 |
| L-Leucine | 12 | 0 | 12 | 12 | 12 |
| L-Lysine-HCl | 14 | 14 | 14 | 14 | 0 |
| L-Methionine | 6 | 6 | 6 | 6 | 6 |
| L-Phenylalanine | 8 | 8 | 8 | 8 | 8 |
| L-Threonine | 8 | 8 | 8 | 8 | 8 |
| L-Tryptophan | 2 | 2 | 2 | 2 | 2 |
| L-Valine | 8 | 8 | 8 | 0 | 8 |
| L-Alanine | 10 | 10 | 10 | 10 | 10 |
| L-Asparagine-H2O | 5 | 5 | 5 | 5 | 5 |
| L-Aspartate | 10 | 10 | 10 | 10 | 10 |
| L-Cystine | 4 | 4 | 4 | 4 | 4 |
| L-Glutamic Acid | 30 | 30 | 30 | 30 | 30 |
| L-Glutamine | 5 | 5 | 5 | 5 | 5 |
| Glycine | 10 | 10 | 10 | 10 | 10 |
| L-Proline | 5 | 5 | 5 | 5 | 5 |
| L-Serine | 5 | 5 | 5 | 5 | 5 |
| L-Tyrosine | 4 | 4 | 4 | 4 | 4 |
| Total L-Amino Acids | 170 | 158 | 162 | 162 | 156 |
|  |  |  |  |  |  |
| Corn Starch | 550.5 | 562.5 | 558.5 | 558.5 | 564.5 |
| Maltodextrin 10 | 125 | 125 | 125 | 125 | 125 |
| Cellulose | 50 | 50 | 50 | 50 | 50 |
|  |  |  |  |  |  |
| Corn Oil | 50 | 50 | 50 | 50 | 18 |
| Mineral Mix S10001 | 35 | 35 | 35 | 35 | 35 |
| Sodium Bicarbonate | 7.5 | 7.5 | 7.5 | 7.5 | 7.5 |
| Vitamin Mix V10001 | 10 | 10 | 10 | 10 | 10 |
| Choline Bitrartrate | 2 | 2 | 2 | 2 | 2 |
|  |  |  |  |  |  |
| Red Dye, FD&C #40 | 0 | 0.025 | 0 | 0.02 | 0.05 |
| Blue Dye, FD&C #1 | 0.05 | 0 | 0 | 0.02 | 0 |
| Yellow Dye, FD&C #5 | 0 | 0.025 | 0.05 | 0.01 | 0 |
|  |  |  |  |  |  |
| Total | 1000.05 | 1000.05 | 1000.05 | 1000.05 | 1000.05 |

| Ingredient (gm） | (-) Met diet | (-) Phe diet | (-) Thr diet | (-) Trp diet |
| --- | --- | --- | --- | --- |
| L-Arginine | 10 | 10 | 10 | 10 |
| L-Histidine-HCl-H2O | 6 | 6 | 6 | 6 |
| L-Isoleucine | 8 | 8 | 8 | 8 |
| L-Leucine | 12 | 12 | 12 | 12 |
| L-Lysine-HCl | 14 | 14 | 14 | 14 |
| L-Methionine | 0 | 6 | 6 | 6 |
| L-Phenylalanine | 8 | 0 | 8 | 8 |
| L-Threonine | 8 | 8 | 0 | 8 |
| L-Tryptophan | 2 | 2 | 2 | 0 |
| L-Valine | 8 | 8 | 8 | 8 |
| L-Alanine | 10 | 10 | 10 | 10 |
| L-Asparagine-H2O | 5 | 5 | 5 | 5 |
| L-Aspartate | 10 | 10 | 10 | 10 |
| L-Cystine | 4 | 4 | 4 | 4 |
| L-Glutamic Acid | 30 | 30 | 30 | 30 |
| L-Glutamine | 5 | 5 | 5 | 5 |
| Glycine | 10 | 10 | 10 | 10 |
| L-Proline | 5 | 5 | 5 | 5 |
| L-Serine | 5 | 5 | 5 | 5 |
| L-Tyrosine | 4 | 4 | 4 | 4 |
| Total L-Amino Acids | 164 | 162 | 162 | 168 |
|  |  |  |  |  |
| Corn Starch | 556.5 | 558.5 | 558.5 | 552.5 |
| Maltodextrin 10 | 125 | 125 | 125 | 125 |
| Cellulose | 50 | 50 | 50 | 50 |
|  |  |  |  |  |
| Corn Oil | 18 | 50 | 50 | 50 |
| Mineral Mix S10001 | 35 | 35 | 35 | 35 |
| Sodium Bicarbonate | 7.5 | 7.5 | 7.5 | 7.5 |
| Vitamin Mix V10001 | 10 | 10 | 10 | 10 |
| Choline Bitrartrate | 2 | 2 | 2 | 2 |
|  |  |  |  |  |
| Red Dye, FD&C #40 | 0.025 | 0 | 0.025 | 0.05 |
| Blue Dye, FD&C #1 | 0.025 | 0.05 | 0 | 0 |
| Yellow Dye, FD&C #5 | 0 | 0 | 0.025 | 0 |
|  |  |  |  |  |
| Total | 1000.05 | 1000.05 | 1000.05 | 1000.05 |

**Supplementary Table 2. Primers used for gene amplification.**

| **Gene** | **Direction** | **Primer sequence 5’→3’** |
| --- | --- | --- |
| *Crh* | F  R | GGAGCCGCCCATCTCTCT  CCGGGCCATTTCCAAGAC |
| *Gcn2* | F  R | CCTGCACCATGAGAACATTG  CTGCCCAGTTCTTCAGTGT |
| *Gapdh* | F  R | TGTGTCCGTCGTGGATCTGA  CCTGCTTCACCACCTTCTTGAT |
| *Agrp* | F  R | GGCCTCAAGAAGACAACTGC  GCAAAAGGCATTGAAGAAGC |
| *Npy* | F  R | CTCCGCTCTGCGACACTAC  AGGGTCTTCAAGCCTTGTTCT |
